# Supplementary material for: Combined Protein- and Ligand-Observed NMR Workflow to Screen Fragment Cocktails against Multiple Proteins: A Case Study Using Bromodomains
Source: Molecules. 2020 Aug 29;25(17):3949. doi: 10.3390/molecules25173949 (PMC7504435; doi:10.3390/molecules25173949)
Supplement: Supplementary file 1 [file molecules-25-03949-s001.pdf]

Supporting Information

Combined Protein- and Ligand-Observed NMR Workflow to Screen Fragment Cocktails against Multiple Proteins: A Case Study Using Bromodomains

Jorden A. Johnson [1], Noelle M. Olson [1], Madison J. Tooker [2], Scott K. Bur [2] and William C.K. Pomerantz [1,*]

[†] Department of Chemistry, University of Minnesota, Minneapolis, Minnesota 55455, United States

[‡] Department of Chemistry, Gustavus Adolphus College, St. Peter, Minnesota 56082, United States

**Table S1**: List of fragments in each mixture purchased from Life Chemicals	3
**Table S2**: PrOF NMR results from part one of the screen.	5
**Table S3**. Screen hits	8
**Table S4**. PBF comparison	12
**Figure S1**: PMI plots	12
**Figure S2**: Circular Dichroism and thermal melts of 5FW and unlabeled *Pf*GCN5	13
**Figure S3**. Overlay of ^{19}F spectra of 5FW *Pf*GCN5 and 15N,5FW *Pf*GCN5.	14
**Figure S4.** ^{1}H-^{15}N HSQC comparison of 5FW and unbaled PfGCN5	15
**Figure S5. Delta/Delta comparison of** ^{1}H-^{15}N HSQC comparison of 5FW and unbaled *Pf*GCN5	17
**Figure S6.** Analysis of the two steps of the screening platform	18
**PrOF NMR titrations of 1-9**	19
**Figure S7.** HPLC trace and MALDI mass spec data for H2A.ZII K7,13ac.	31
**Figure S8.** Titration of GSK4027 with *Pf*GCN5	31
**Figure S9.** Stacked spectra of various 5FW labeled bromodomains.	33
**Table S5.** Competition ^{1}H CPMG NMR of **9**.	34
**Figure S10.** Competition ^{1}H CPMG NMR of **9** raw data	34
**Figure S11.** ^{1}H -^{15}N HSQC titration of **9** with PfGCN5	35
**Figure S12.** Δδ for each residue for the ^{1}H -^{15}N HSQC titration of **9** with *Pf*GCN5	38
**Figure S13.** Titration isotherms for the ^{1}H -^{15}N HSQC titration of **9** with *Pf*GCN5	39
**Figure S14.** ^{1}H -^{15}N HSQC titration of H2A.Z II K7,13ac with *Pf*GCN5	41
**Figure S15.** Δδ for each residue for the ^{1}H -^{15}N HSQC titration of H2A.Z II K7,13ac with *Pf*GCN5.	42
**Figure S16.** ^{1}H -^{15}N HSQC titration of **9** with a saturating concentration of H2A.Z II K7,13ac with *Pf*GCN5	43
**Figure S17.** Δδ for each residue for the ^{1}H -^{15}N HSQC titration of **9** with a saturating concentration of H2A.Z II K7,13ac with *Pf*GCN5	45
**Figure S18.** ^{1}H -^{15}N HSQC titration of GSK4207 with *Pf*GCN5	47
**Figure S19.** Δδ for each residue for the ^{1}H -^{15}N HSQC titration of GSK4027 with *Pf*GCN5	48
**Figure S20.** FT map of *Pf*GCN5	49
**Figure S21.** 5FW PfGCN5 titration with bromosporine	50
**Figure S22.** PrOF NMR of 5FW-*Pf*GCN5 and W1375F 5FW-*Pf*GCN5	50
**Table S6.** Chemical shifts and resonance width of 5FW labeled bromodomain when tested alone or in the presence of another 5FW labeled bromodomain.	51

Table S1: List of fragments in each mixture purchased from Life Chemicals

Mixture number	Frag. 1	Frag. 2	Frag.3	Frag. 4	Frag. 5
1	F0917-7549	F0119-0013	F0007-0920	F0111-0053	F0138-4744
2	F1011-0339	F0840-0009	F0193-0249	F0266-1236	F0326-1084
3	F1727-0265	F1170-0153	DMSO	F0413-0005	F0414-0069
4	DMSO	F1294-0014	F2147-1744	F0417-2042	F0550-0006
5	F2147-1414	DMSO	F2147-1746	F0777-0005	F0777-0038
6	DMSO	F2158-1186	F2158-1528	F0848-0336	F0863-0275
7	F2196-0128	F2185-0055	F2189-0042	F0882-2747	F1019-0073
8	F0840-0018	F0916-0420	F2196-0060	F1371-0182	F1386-0087
9	F1294-0083	F1905-0231	F2196-0115	F1467-0819	F1826-0009
10	F1904-0061	F1905-0068	F2328-0112	F1826-0017	F1838-0001
11	F1618-0607	F1905-0298	F2513-0609	F1847-0012	F1852-0063
12	F1905-0301	F1905-0308	F3305-0600	F1905-0021	F1905-0303
13	F1905-0309	F1905-0329	F5860-1601	F1905-0374	F1905-0493
14	F1905-0335	F2124-0592	F1905-0677	F1905-3201	F1924-0041
15	F1905-0369	F2124-0634	F1905-6475	F1905-6607	F1905-6474
16	F1905-0401	F2147-1745	F1967-0429	F1967-0973	F2145-0393
17	F1905-0501	F6438-0300	F1983-0015	F2070-1293	F2087-0068
18	F1905-0503	F6438-2140	F2130-0041	F2135-0967	F2145-0303
19	F1905-0651	F8885-8052	F1967-0977	F2158-0001	F2146-0113
20	F1905-6593	F8880-7458	F2147-0164	F2147-0955	DMSO
21	F1905-6609	F9995-1074	F2145-0685	F2158-0166	F2158-1788
22	F1906-0103	F2158-1792	F2167-0225	DMSO	F2666-0003
23	F1907-0005	F2174-0004	DMSO	F2199-0031	F2545-0414
24	F1909-0045	F2189-0385	F2189-0401	F2185-0095	F2256-0052
25	F1912-0151	F2477-0016	F2545-0082	F2185-0148	F2590-0203
26	F1957-0076	DMSO	F2616-0737	F2879-1558	F2764-0088
27	F1957-0096	F2792-0286	F2823-0004	F2879-0168	F2879-0206
29	F2135-1033	F3176-0122	F5857-6331	DMSO	DMSO
30	F2142-0034	F3260-0714	F3260-0726	F3266-0084	F3308-2767
31	F2145-0188	F3394-0174	F5003-0140	F5003-0141	F5032-0036
32	F2145-0759	F5033-3307	F5033-3510	F5036-0073	F5061-0129
33	F2145-0842	F5061-0204	F5079-0044	F5103-0363	F5139-0391
34	F2145-0854	F5140-0384	F5223-0061	F5303-0092	F5306-0558
35	F2145-0905	F5330-0648	F5496-0578	F5626-0027	F5626-0058
36	F2147-0086	F5657-0074	F5831-9937	F5832-0077	F5835-0012
37	F2147-0202	F5852-0822	F5852-1840	F5852-2037	F5857-1897
38	F2147-0796	F5857-4931	F5857-5714	F5857-5966	F5857-5976
39	F2147-0815	F5857-6022	F5857-6033	F3257-0390	F5857-6355
40	F2147-0823	F5857-6411	F5857-6432	F5857-7085	F5857-8596

41	F2147-0844	F5860-4337	F5882-4181	F5964-0063	F5964-0234
42	F2147-0847	F6031-1898	F5965-0049	F5965-0078	F5971-0153
43	DMSO	F5964-0253	F6057-0058	F6089-9654	F6089-9657
44	F2147-1469	F6089-9693	F6089-9714	F6089-9738	F6089-9749
45	F2147-1517	F6089-9751	F6089-9752	F6089-9758	F6089-9760
46	F2147-1605	F6089-9775	F6089-9807	F6089-9809	F6089-9813
47	F2147-1723	F6089-9815	F6089-9817	F6089-9819	F6089-9820
48	F2158-0132	F6089-9823	F6089-9824	F6089-9832	F6089-9837
49	F2158-0871	F6089-9840	F6089-9846	F6089-9854	F6089-9869
50	F2158-1471	F6125-0675	F6125-2998	F6125-3005	F6142-0007
51	DMSO	F6142-0022	F6170-0022	F6172-0211	F6184-0396
52	DMSO	F6186-0476	F6186-1443	F6190-0015	F6190-0346
53	F2184-0025	F6190-0427	F6190-0464	F6190-0472	F6190-0594
54	F2184-0232	F6190-1498	F6194-0017	F6194-0225	F6201-0341
55	F2184-0233	F6210-1005	F6212-0099	F6213-0268	F6226-0250
56	F2185-0035	F6245-0110	F6266-0028	DMSO	F6266-0359
57	F2185-0036	F6279-0683	F6293-0029	F6350-0181	F6353-5554
58	F2185-0134	F6353-5562	F6353-5787	F6353-6020	F6356-0896
59	F2185-0145	F6356-1089	F6356-1113	F6356-1114	F6356-1266
60	F2185-0182	DMSO	F6414-1220	F6416-8778	F6416-8945
61	F2158-1682	F6416-9158	F6416-9173	F6420-0009	F6420-0341
62	F2165-0032	F6420-0570	F6420-0571	F6435-0630	F6435-1434
63	F2167-0008	F6435-1461	F6435-5410	F6435-5442	F6435-9142
64	F2167-0028	F6436-0711	F6436-2326	F6436-2818	F6436-3945
65	F2167-0202	F6437-0082	F6437-0094	F6438-0003	F6438-0052
66	F2167-1047	DMSO	F6438-0398	F6438-0663	F6438-0735
67	F2167-1073	F6438-0736	F6438-0864	F6438-0865	F6446-0528
68	F2167-1224	F6438-1296	F6438-1301	F6438-1303	F6438-1669
69	F2167-1787	DMSO	F6439-2685	F6439-3453	F6440-2337
70	DMSO	F6440-2340	F6440-3066	F6440-2316	F6441-3745
71	DMSO	F6441-4791	DMSO	F6442-0004	F6442-1592
72	F2167-2349	F6442-1953	F6442-1971	F6442-1973	F6443-4297
73	F2167-2352	F6443-6532	F6443-7576	F6443-8049	F6444-2113
74	F2167-2773	F6444-2124	F6444-2578	DMSO	F6445-1309
75	F2169-0271	F6448-0519	F6448-1031	F6448-1373	DMSO
76	F2185-0199	F2186-0306	F6448-1618	F6448-1757	F6451-0004
77	F2186-0569	F2187-2219	F6451-0218	F6451-0526	F6451-0740
78	F2189-0578	F2189-0686	F6451-0766	F6451-0768	F6452-0526
79	F2189-0794	F2189-1059	F6452-1288	F6454-0218	F6464-1254
80	F2196-0126	F3308-2774	F6471-0004	F6472-0515	F6472-0702
81	F5017-1352	F2196-0139	F6472-0733	F6473-4843	F6473-8004
82	F5831-7030	F2199-0308	F6475-1586	F6475-4585	F6475-5596
83	F2199-0320	F2616-0581	F6475-6115	F6475-7111	F6475-7791

84	F2196-0134	F5016-0010	F6478-3785	F6479-2652	F6481-0067
85	F2196-0136	F5831-6427	F6481-1232	F6481-1593	F6481-2746
86	F2199-0137	DMSO	F6481-3912	F6481-3967	F6481-4105
87	DMSO	F6031-0286	F6481-4229	F6481-4431	F6482-2041
88	F6031-1161	F6108-0073	F6482-2049	F6482-2052	F6482-2054
89	F6089-9658	F6031-1283	F6482-2061	F6482-2216	F6482-2320
90	F6125-2175	DMSO	F6482-2327	F6482-2328	F6487-0659
91	DMSO	F6178-7462	F6488-2366	F6489-0852	F6489-0905
92	DMSO	F6353-6069	F6492-0420	F6497-0456	F6497-0780
93	F8888-7363	F6353-6536	F6497-5647	F6502-9095	F6502-9157
94	F9995-2403	F6438-1468	F6513-5793	F6518-4331	F6521-1551
95	F6492-0614	DMSO	F6521-3312	F6521-3559	F6521-7235
96	F6541-0798	DMSO	F6521-7973	F6521-8993	F6521-9485
97	F6545-0770	F9995-2553	F6525-0192	F6530-0044	F6540-0945
98	F8888-6524	F8880-2936	F6540-4005	F6540-4126	F6540-4194
99	DMSO	F6353-7315	F6540-4336	F6540-4349	F6541-4533
100	F9995-2479	F6353-6303	F6541-4551	F6544-0539	F9995-0012

Table S2: PrOF NMR results from part one of the screen.

Mixture Number	BPTF Δδ (ppm)	*Pf*GCN5 (WPF shelf) Δδ (ppm)	*Pf*GCN5 Δδ (ppm)	Bind BPTF ?[1]	Bind *Pf*GCN 5? [1]	Bind Both Proteins?	Bind Neither Protein?	Number of BPTF hits from CPMG[2]	Number of *Pf*GCN5 hits from CPMG[2]
1	0.039	0.022	0.005	yes				0[$]	-
2	0.135	0.010	0.004	yes				0[#]	-
3	0.020	0.003	0.000				yes	-	-
4	0.031	0.001	0.010	yes				1	-
5	0.187	0.053	0.096			yes		2	1
6	0.075	0.061	0.024			yes		3	*
7	0.064	0.026	0.018	yes				3	3[&]
8	0.005	0.007	0.011				yes	-	-
9	0.007	0.008	0.001				yes	-	-
10	0.009	0.019	0.005				yes	-	-
11	0.054	0.012	0.014	yes				4	-
12	0.009	0.006	0.005				yes	-	-
13	0.006	0.005	0.002				yes	-	-
14	0.045	0.072	0.001			yes		0[$]	0[$]
15	0.012	0.006	0.000				yes	-	-
16	0.004	0.059	0.033		yes			-	*
17	0.026	0.040	0.020		yes			-	2
18	0.013	0.013	0.015				yes	-	-
19	0.179	0.006	0.023	yes				1	-

20	0.008	0.046	0.020		yes			-	3
21	0.023	0.058	0.018		yes			-	3
22	0.283	0.149	0.015			yes		2	2
23	0.010	0.001	0.019				yes	-	-
24	0.295	0.084	0.010			yes		1	1
25	0.057	0.030	0.017			yes		2	*
26	0.028	0.020	0.022				yes	-	-
27	0.020	0.009	0.027				yes	-	-
29	0.239	0.131	0.004			yes		2	1
30	0.469	0.169	0.093			yes		%	%
31	0.298	0.034	0.007			yes		1	1
32	0.103	0.463	0.046			yes		3	2
33	0.038	0.009	0.008	yes				1	-
34	NA	NA	NA	NA	NA	NA	NA	-	-
35	0.038	0.081	0.011			yes		3	3
36	0.031	0.012	0.015	yes				1	-
37	0.025	0.018	0.016				yes	-	-
38	0.026	0.032	0.017		yes			0[$]	-
39	0.026	0.027	0.025				yes	-	-
40	0.013	0.024	0.020				yes	-	-
41	0.007	0.012	0.001				yes	-	-
42	0.008	0.003	0.037				yes	-	-
43	0.020	0.015	0.018				yes	-	-
44	0.006	0.025	0.019				yes	-	-
45	0.000	0.001	0.000				yes	-	-
46	0.014	0.274	0.023		yes			-	!
47	0.031	0.026	0.024	yes				2	-
48	0.005	0.008	0.018				yes	-	-
49	0.024	0.010	0.022				yes	-	-
50	0.051	0.011	0.020	yes				3	-
51	0.001	0.005	0.017				yes	-	-
52	0.234	0.208	0.210			yes		-	!
53	0.030	0.017	0.028	yes				4	-
54	0.020	0.019	0.019				yes	-	-
55	0.010	0.003	0.020				yes	-	-
56	0.001	0.009	0.017				yes	-	-
57	0.016	0.015	0.009				yes	-	-
58	0.030	0.039	0.020		yes			-	2
59	0.028	0.032	0.021		yes			-	0[$]
60	0.019	0.012	0.022				yes	-	-
61	0.028	0.022	0.022				yes	-	-
62	0.005	0.003	0.023				yes	-	-
63	0.041	0.028	0.021	yes				0	-

64	0.025	0.016	0.018				yes	-	-
65	0.019	0.024	0.017				yes	-	-
66	0.030	0.044	0.019		yes			-	*
67	0.021	0.057	0.023		yes			-	1
68	0.001	0.207	0.021		yes			-	2
69	0.004	0.023	0.043		yes			-	*
70	0.022	0.020	0.007				yes	-	-
71	0.000	0.011	0.004				yes	-	-
72	0.134	0.206	0.023			yes		1	3
73	0.001	0.000	0.006				yes	-	-
74	0.015	0.022	0.007				yes	-	-
75	0.019	0.004	0.007				yes	-	-
76	0.117	0.031	0.006			yes		*	3
77	0.001	0.004	0.009				yes	-	-
78	0.013	0.014	0.005				yes	-	-
79	0.069	0.033	0.011			yes		2	2
80	0.006	0.023	0.004				yes	-	-
81	0.068	0.084	0.005			yes		*	1
82	0.008	0.021	0.002				yes	-	-
83	0.142	0.019	0.005	yes				0[#]	-
84	0.003	0.183	0.004		yes			-	1
85	0.008	0.013	0.013				yes	-	-
86	0.026	0.079	0.000		yes			-	0
87	0.002	0.018	0.001				yes	-	-
88	0.002	0.051	0.002		yes			-	1
89	0.001	0.011	0.002				yes	-	-
90	0.003	0.006	0.004				yes	-	-
91	0.016	0.000	0.006				yes	-	-
92	0.202	0.259	0.022			yes		1	1
93	0.000	0.007	0.009				yes	-	-
94	0.067	0.030	0.044			yes		2	5
95	0.012	0.007	0.004				yes	-	-
96	0.007	0.008	0.007				yes	-	-
97	0.019	0.016	0.000				yes	-	-
98	0.043	0.009	0.001	yes				0[$]	-
99	0.031	0.021	0.002	yes				3	-
100	0.028	0.020	0.005				yes	-	-
Average	0.049	0.043	0.017	-	-	-	-	-	-

[1]The prior PrOF NMR hit criteria of $\Delta\delta \geq 0.03$ ppm was used.[1] This was a conservative cutoff as the average $\Delta\delta$ for the WPF shelf resonances for BPTF and *Pf*GCN5 are 0.049 (*s* = 0.079 ppm) and 0.043 (*s* = 0.070 ppm.) [2]A resonance decrease of ≥ 20% was considered a ^{1}H CPMG NMR hit.[$] = no hits but the $\Delta\delta < 0.05$ indicating a probable false positive; [#] = no hits but the $\Delta\delta > 0.05$ indicating a likely false ^{1}H CPMG

false negative or aggregate binding in the PrOF NMR assay; & = PfGCN5 was tested although not a PrOF NMR mixture hit, hits were found indicating a PrOF NMR false negative; * = mixture not tested; ! = data was unusable due to poor water suppression.

Table S3. Screen hits

Fragment	Mixture number	Structure	Bind BPTF? (K_d in µM)[2]	BPTF CPMG % decrease	Bind PfGCN5? (K_d in µM)[2]	PfGCN5 CPMG % decrease	Bind BRD4? (K_d in µM)	PBF
F0863-0257 **(4)**	6	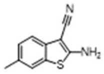	Y	100 (NB)	N	-	N	0.097
F1905-0651	19	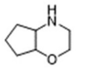	Y	75	N	-	N	0.186
F6190-0464	53	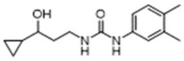	Y	67	N	-	N	0.374
F6142-0007 **(5)**	50	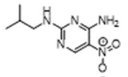	Y	65 (NS)	N	-	N	0.200
F0550-0006	4	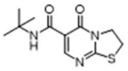	Y	63	N	-	N	0.199
F5036-0073	32	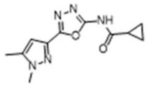	Y	55	N	-	Y (1200)	0.150
F0777-0005	5	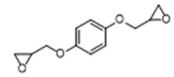	Y	54	N	-	N	0.247
F2590-0203	25	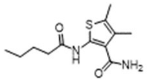	Y	53	N	-	N	0.141
F6190-0464	53	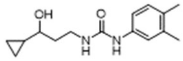	Y	50	N	-	N	0.374
F6540-4349	99	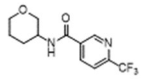	Y	50	N	-	N	0.489
F6541-4533	99	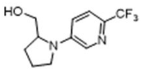	Y	48	N	-	N	0.678
F6190-0594	53	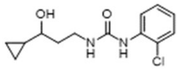	Y	48	N	-	N	0.273
F1905-0298	11	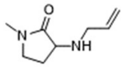	Y	46	N	-	N	0.268
F2513-0609	11	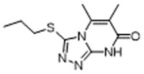	Y	46	N	-	N	0.001
F2158-1471	50	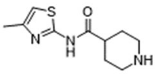	Y	40	N	-	N	0.288
F1852-0063	11	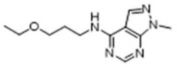	Y]	38	N	-	N	0.001

F6190-0472	53	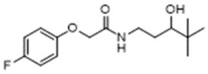	Y	36	N	-	N	0.327
F1847-0012	11	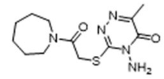	Y	36	N	-	N	0.415
F2477-0016	25	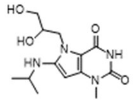	Y	34	N	-	N	0.481
F0848-0336	6	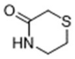	Y	33	NT	-	N	0.177
F6125-0675	50	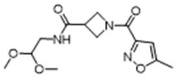	Y	32	N	-	N	0.444
F2145-0759	32	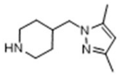	Y	29	N	-	N	0.453
F2158-1186	6	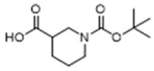	Y	29	NT	-	N	0.410
F5657-0074	36	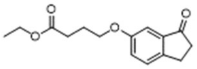	Y	27	N	-	Y (80)	0.002
F5139-0391 **(6)**	33	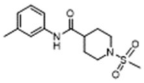	Y (540)	25	N	-	N	0.581
F2135-1033	29	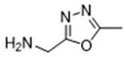	Y	23	N	-	N	0.271
F2147-1723	47	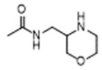	Y	23	N	-	N	0.219
F6353-7315	99	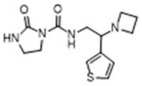	Y	23	N	-	N	0.436
F6089-9820	47	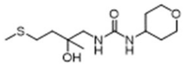	Y	23	N	-	N	0.436
F6482-2052 **(1)**	88	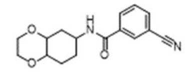	N	-	Y (NS)	100	N	0.485
F5033-3307 **(2)**	32	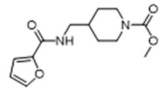	N	-	Y (360)	100	Y (450)	0.405
F6438-1296 **(3)**	68	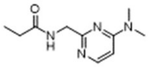	N	-	Y (NS)	82	N	0.439
F6521-1551	94	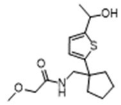	N	-	Y	70	N	0.613
F6438-0864	67	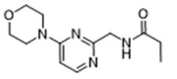	N	-	Y	67	N	0.416
F6518-4331	94	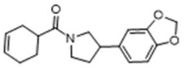	N	-	Y	58	N	0.643
F6481-0067	84	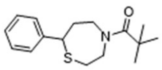	N	-	Y	53	N	0.718

F6438-1301	68	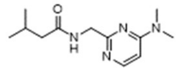	N	-	Y	50	N	0.500
F2147-0955	20	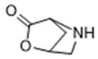	N	-	Y	50	N	0.381
F1905-6609	21	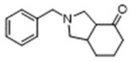	N	-	Y	50	N	0.430
F1983-0015	17	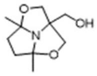	N	-	Y	50	N	0.623
F6438-0300	17	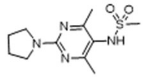	N	-	Y	42	N	0.459
F9995-1074	21	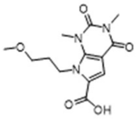	N	-	Y	41	N	0.425
F2158-1788	21	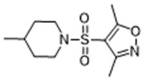	N	-	Y	40	N	0.938
F6353-5787	58	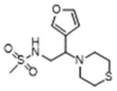	N	-	Y	40	N	0.748
F6521-1551	94	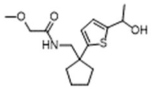	N	-	Y	32	N	0.613
F6448-1757	76	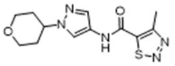	N	-	Y	35	N	0.326
F6442-1973	72	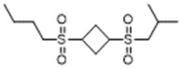	N	-	Y	26	N	0.629
F6451-0004	76	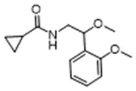	N	-	Y	26	N	0.717
F6356-0896	58	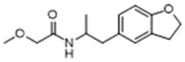	N	-	Y	25	Y (NC)	0.643
F6473-4843	81	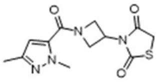	N	-	Y	25	Y(NC)	0.838
F8880-7458	20	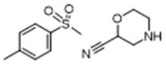	N	-	Y	24	N	0.213
F6442-1971	72	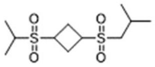	N	-	Y	22	N	0.705
F1905-6593	20	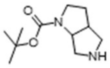	N	-	Y	21	N	0.559
F6448-1757	76	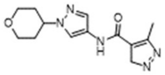	N	-	Y	20	Y (210)	0.326
F0777-0038	5	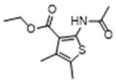	Y	100	Y	100	Y (820)	0.385
F6497-0456	92	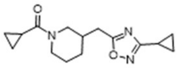	Y	100	Y	100	N	0.715

F3176-0122	29	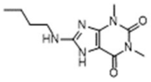	Y	80	Y	77	Y (2500)	0.001
F5626-0027	35	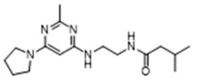	Y	77	Y	75	Y	0.284
F6513-5793	94	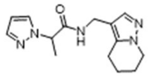	Y	72	Y	49	N	0.525
F6464-1254	79	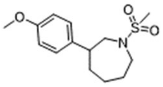	Y	69	Y	33	N	0.635
F6438-1468	94	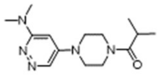	Y	62	Y	100	Y	0.379
F5496-0578	35	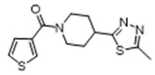	Y	55	Y	83	N	0.655
F0882-2747	7	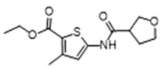	Y	54	Y	63	Y (660)	0.349
F2158-1792	22	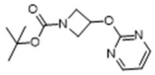	Y	54	Y	68	N	0.481
F2666-0003	22	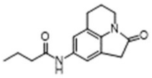	Y	54	Y	100	Y (840)	0.284
F2256-0052 **(8)**	24	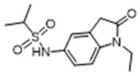	Y (720)	53	Y (>10000)	38	Y (24)	0.698
F5003-0140 / F5003-0141 *	31	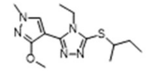	Y	52	Y	36	Y	0.5146906, 0.65689
F5626-0058	35	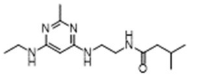	Y	50	Y	47	Y (NC)	0.141
F6443-4297 **(7)**	72	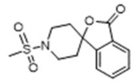	Y(> 1000)	49	Y (150)	100	N	0.397
F5033-3510	32	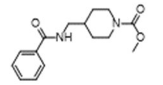	Y	42	Y	68	N	0.531
F2189-0042	7	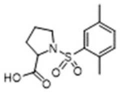	Y	35	Y	32	N	0.776
F6452-1288	79	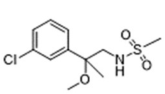	Y	32	Y	57	N	0.516
F2185-0055	7	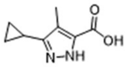	Y	31	Y	29	N	0.169
F3308-2767‡ **(9)**	30	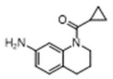	Y (180)	100	Y (17)	100	Y (50)	0.222
Average	-	-	-	51		56		0.419

[2]A resonance decrease of ≥ 20% was considered a ^{1}H CPMG NMR hit. NC = noncompetitive with JQ1 in a ^{1}H CPMG competition experiment; * = resonances overlap in ^{1}H CPMG assay; ‡ = mixture was not tested in the ^{1}H

CPMG assay but the fragment has been found to bind previously. NS = Does dependent binding that does not saturate.

Table S4: PBF comparison.

PBF Distribution Comparison	Same Distribution	p-value (MWW)
Library vs All Hits	yes	0.093
Library vs BPTF Hits	no	4.75 *10-6
Library vs PfGCN5 hits	yes	0.0291
Library vs Hits for both BPTF and PfGCN5	yes	0.7645
BPTF Hits vs PfGCN5	no	1.443* 10-5
BPTF Hits vs Hits binding both BPTF and PfGCN5	yes	0.0132
BPTF hits vs All hits	yes	0.00635
PfGCN5 Hits vs Hits for both BPTF and PfGCN5	yes	0.122
PfGCN5 Hits vs All hits	yes	0.0107

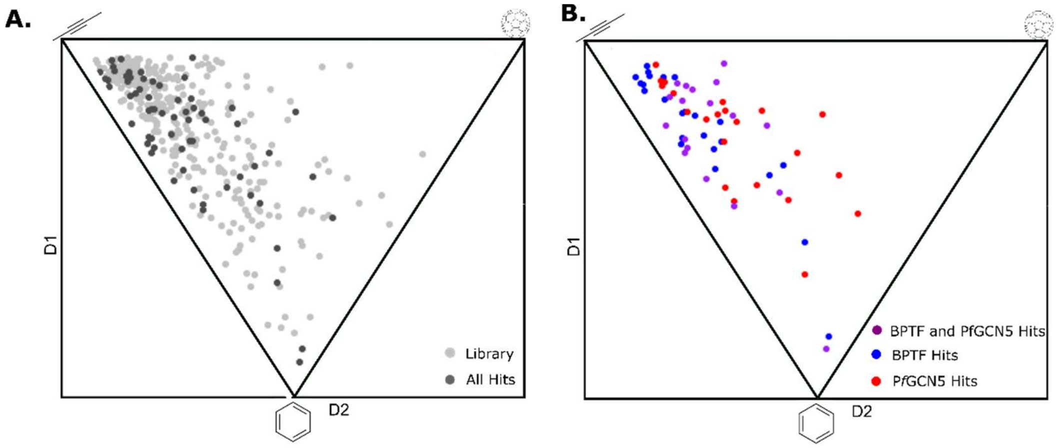

Figure S1. Principle moment of inertia plots comparing the 3D-character of A) overall library and hits and B) the hits selective for BPTF or PfGCN5 and the hits that bound both proteins.

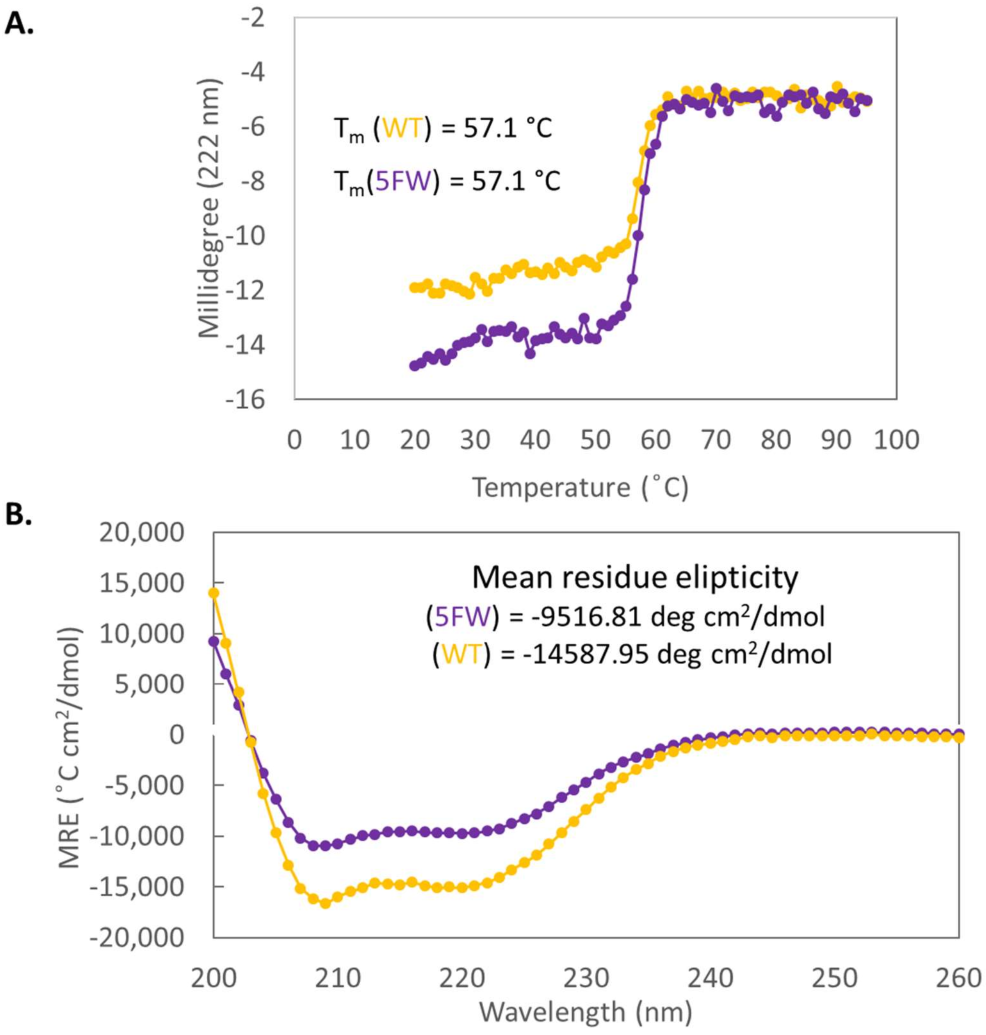

Figure S2: 5FW and unlabeled *Pf*GCN5 circular dichroism secondary structure comparison. A) Thermal melt measured at 222 nm. B) Circular dichroism trace and molar residue ellipticity.

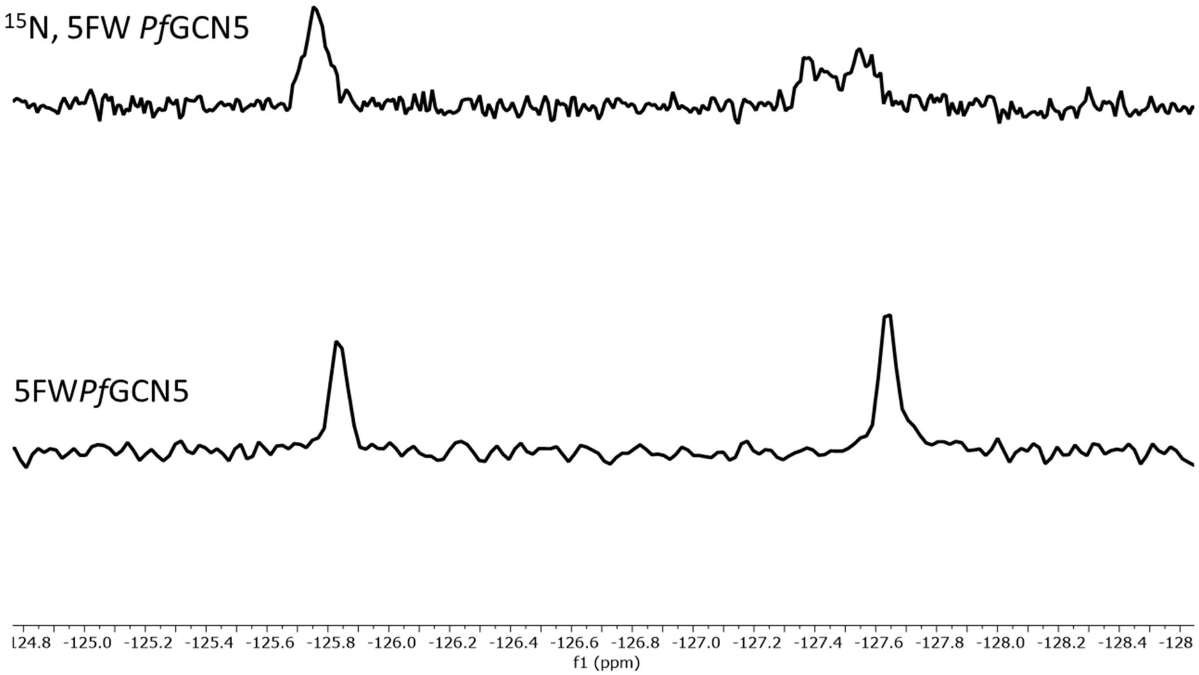

Figure S3: Overlay of ^{19}F spectra of 5FW *Pf*GCN5 and ^{15}N,5FW *Pf*GCN5. The upfield resonance at -127.4-.6 ppm indicates a heterogeneous population of labeled protein. See **figure S4** for note on fluorine incorporation.

A.

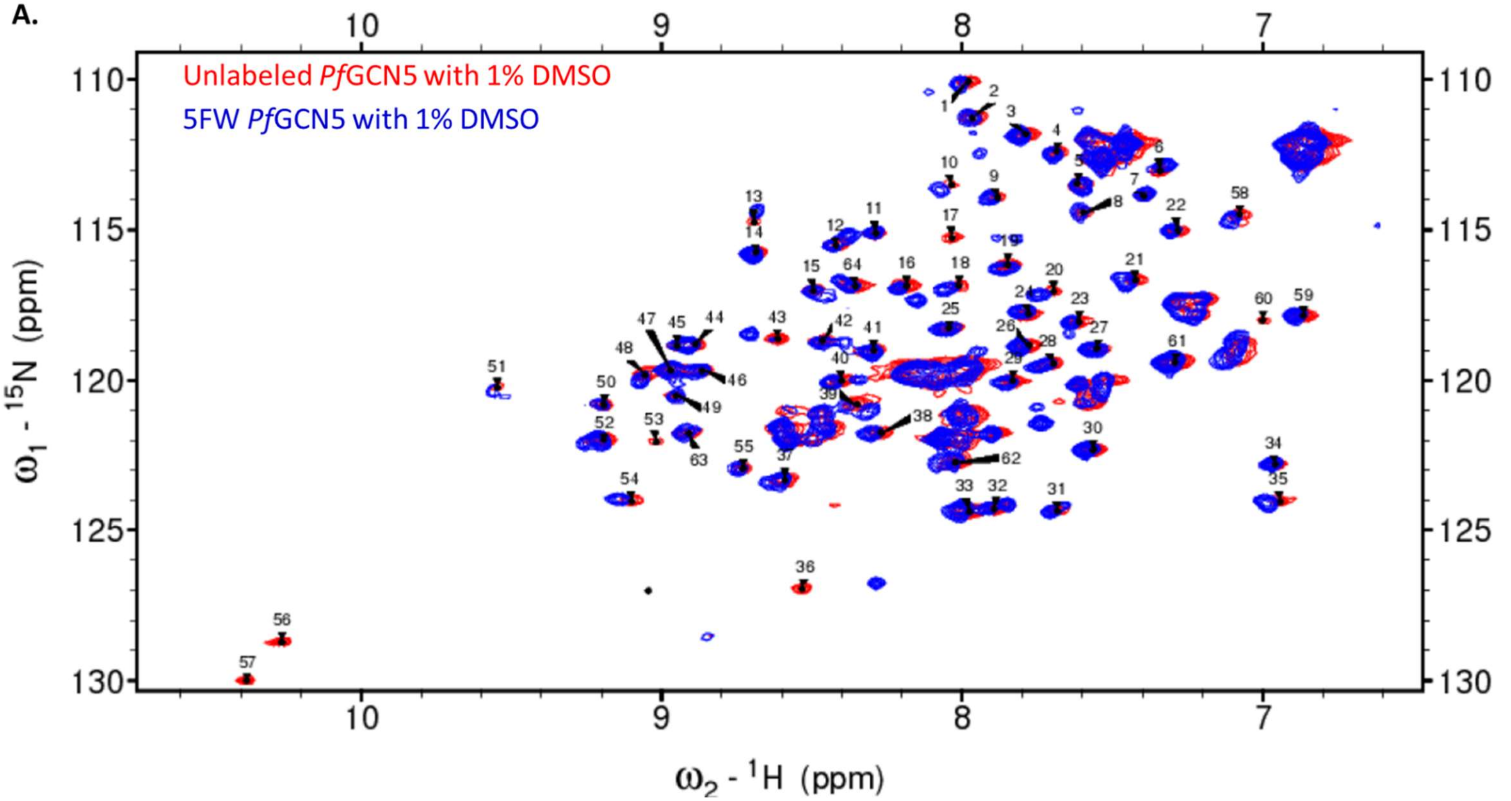

Unlabeled *Pf*GCN5 with 1% DMSO
5FW *Pf*GCN5 with 1% DMSO
ω_1 - ^{15}N (ppm)
ω_2 - ^{1}H (ppm)
10
9
8
7
110
115
120
125
130

B.

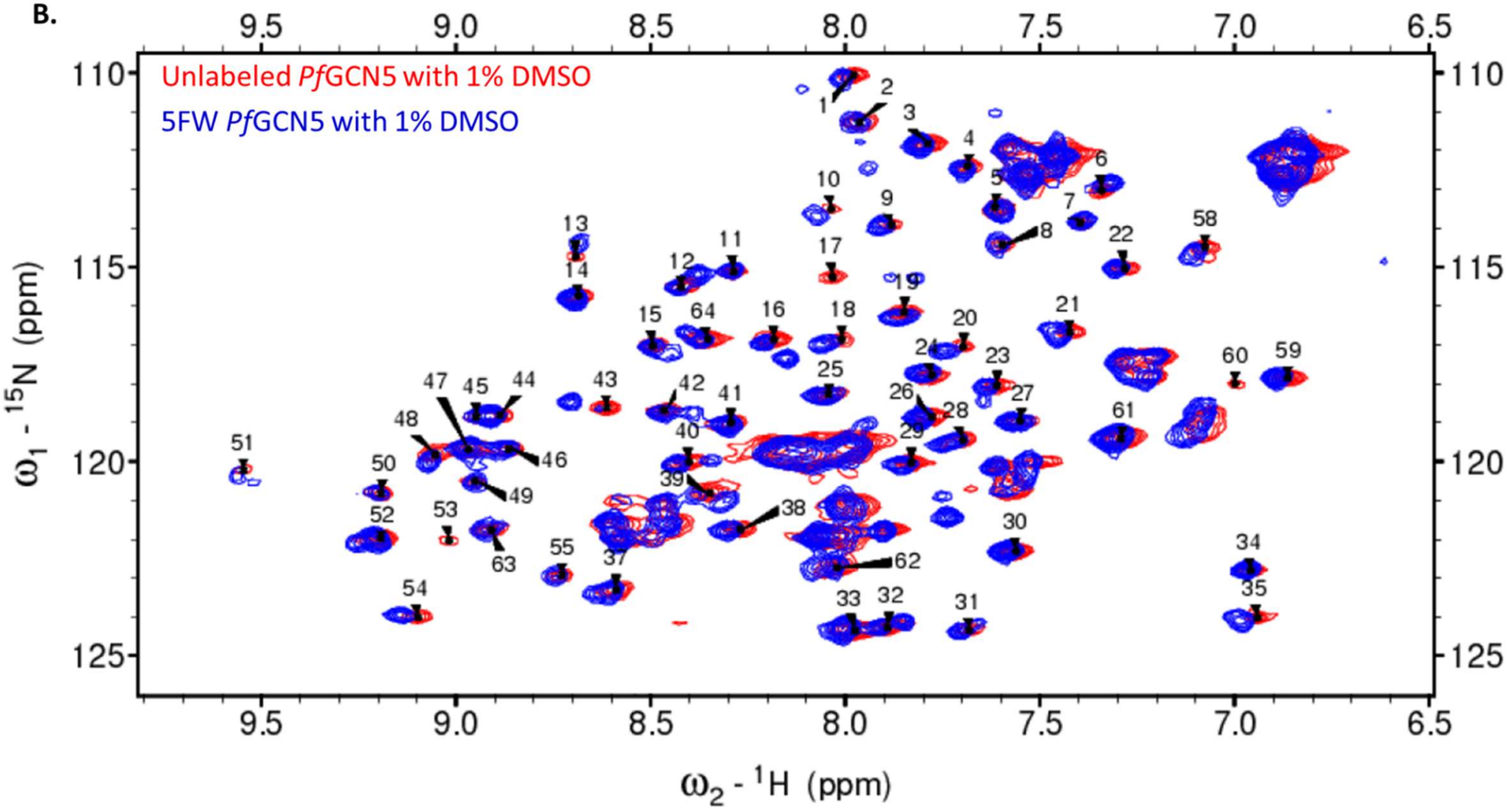

Figure S4. ^{15}N,5FW *Pf*GCN5 and unlabeled *Pf*GCN5 ^{1}H-^{15}N HSQC overlay. A) Full spectra. B) Zoomed in portion of the spectrum. The ^{15}N,5FW *Pf*GCN5 spectrum does not have the tryptophan indole ^{15}N-^{1}H cross peak (*56*, *57*) because non-^{15}N enriched 5-fluoroindole was converted to 5FW via the Shikimate pathway during the protein expression resulting in only the amide nitrogen or tryptophans being ^{15}N labeled. The lack of the ^{15}N-^{1}H cross peak (*56*, *57*) indicates that the ^{15}N,5FW *Pf*GCN5 is successfully ^{19}F labeled.

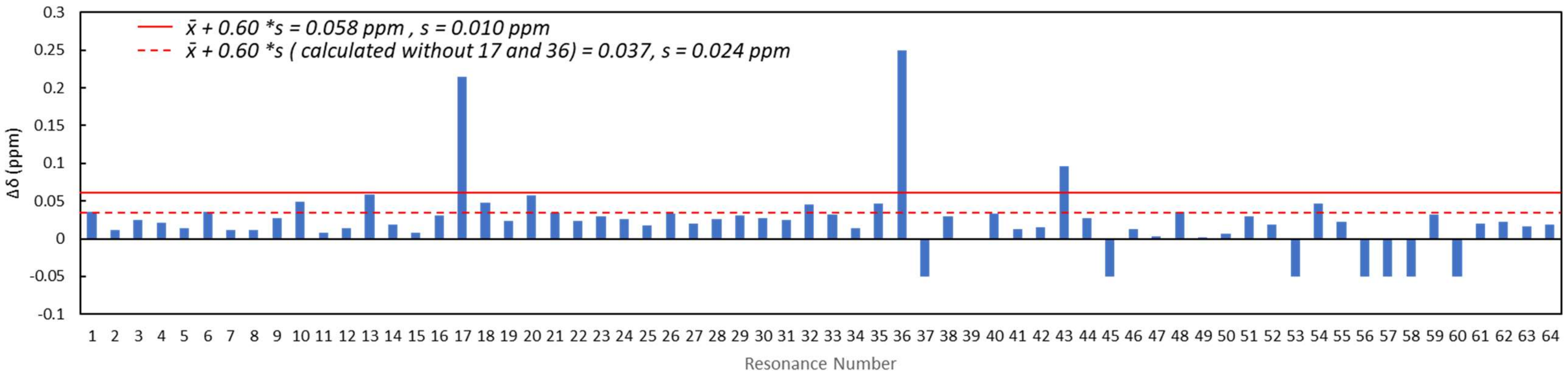

Figure S5. A) Δδ of each resonance between ^{15}N,5FW PfGCN5 and ^{15}N *Pf*GCN5. -0.05 are residues that disappear. Horizontal lines represent the cutoffs of 60% of one standard deviation of the mean and 63% of one standard deviation of the mean omitting resonances *17* and *36* from the calculation. These two resonances may correspond to the two fluorinated tryptophans, however the assignment has not been confirmed.

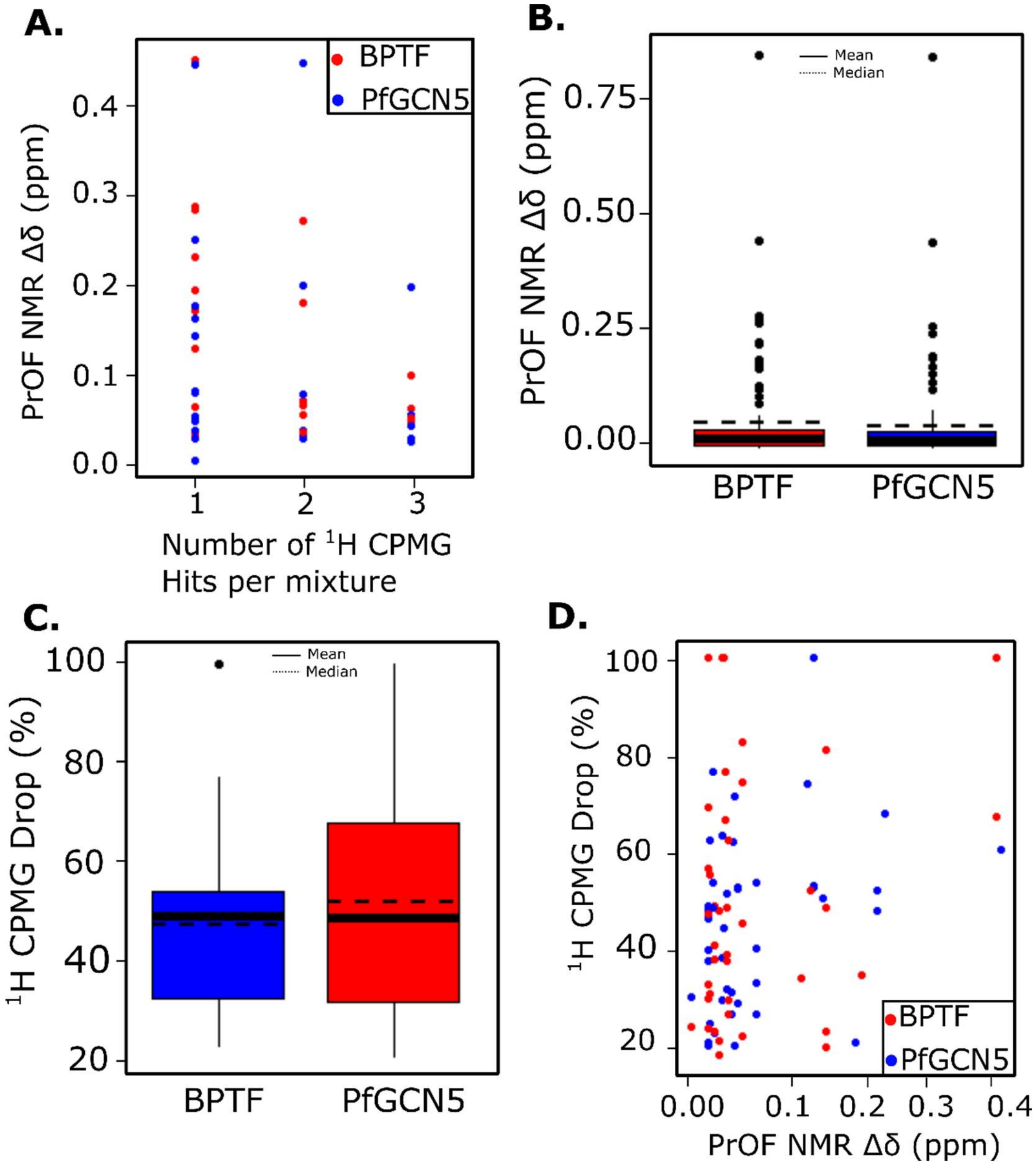

Figure S6. Analysis of the two steps of the screening platform. A) Strip plot showing the number of hits per mixture for the first PrOF NMR step in the screen. B) Box plot showing the distribution of Δδ for the PrOF NMR mixtures from step one of the screen. C) Boxplot of the distribution of % decrease in resonance intensity for the hits determined in step two of the screen, ^{1}H CPMG deconvolution. D) Comparison of the % decrease of resonance intensity of the hits vs the Δδ of the PrOF NMR mixture the hits came from.

PrOF NMR titration of **1** with *Pf*GCN5.

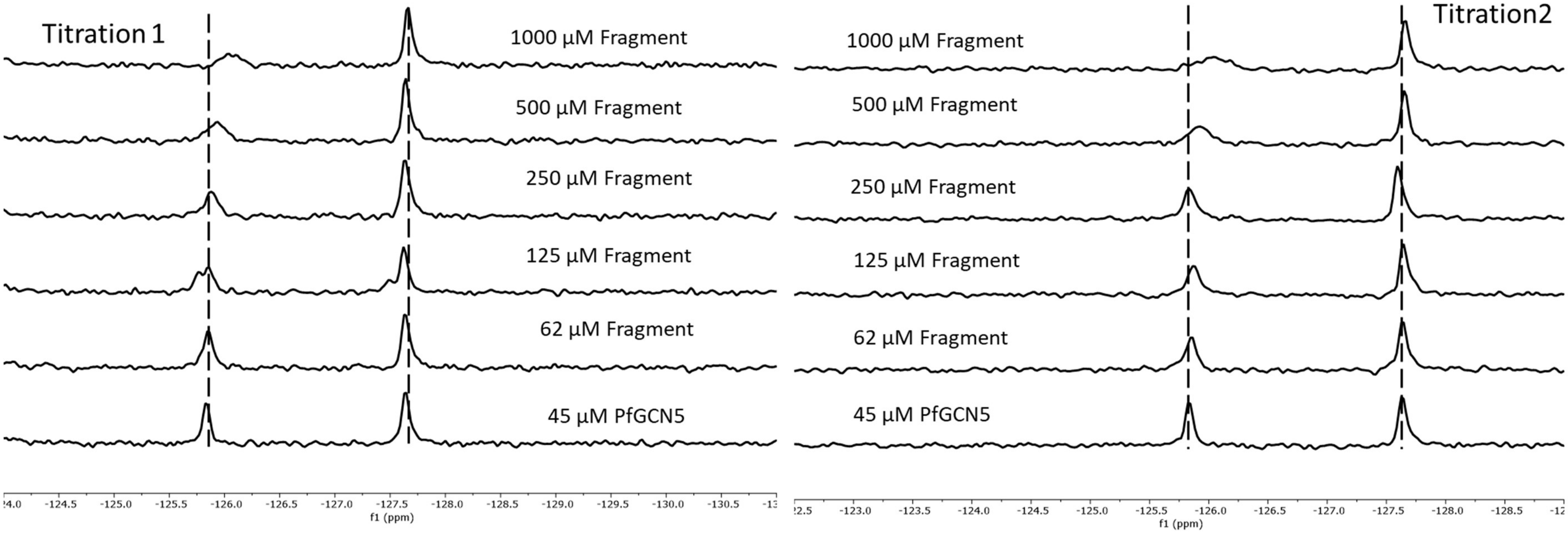

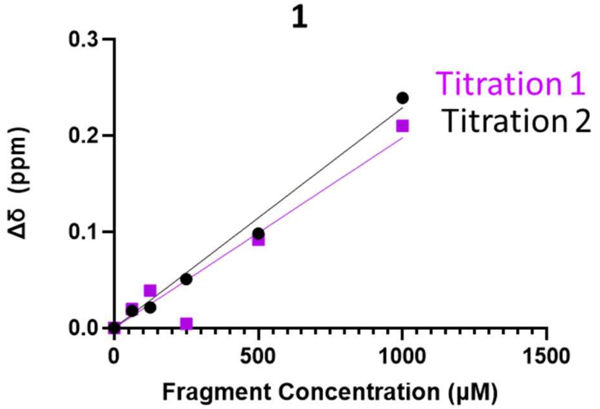

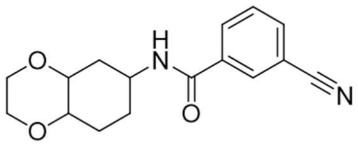

PrOF NMR titration of **2** with *Pf*GCN5.

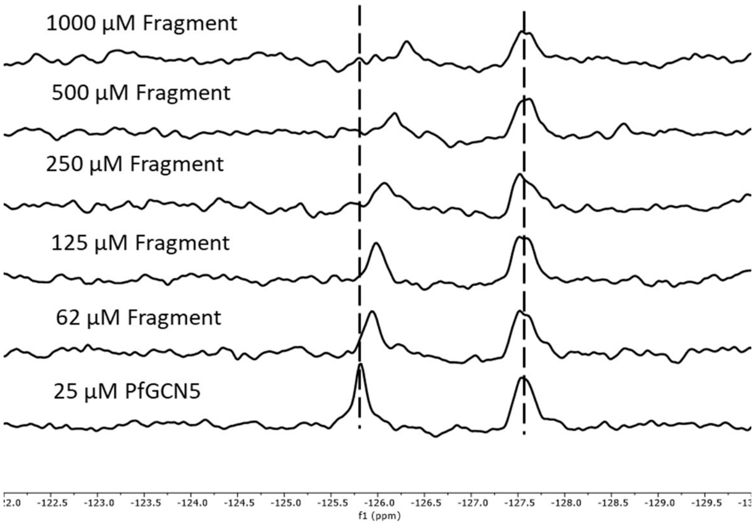

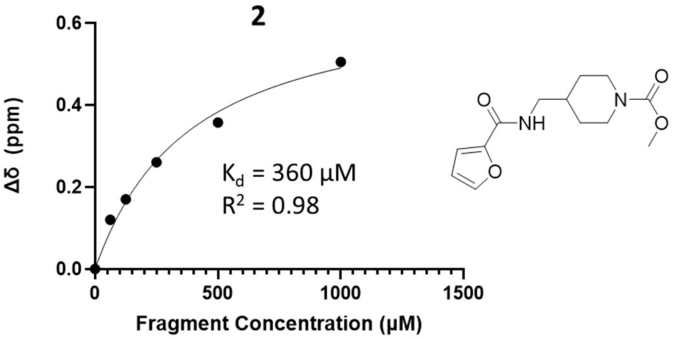

PrOF NMR titration of **3** with *Pf*GCN5. Dose-dependent binding is observed but does not saturate.

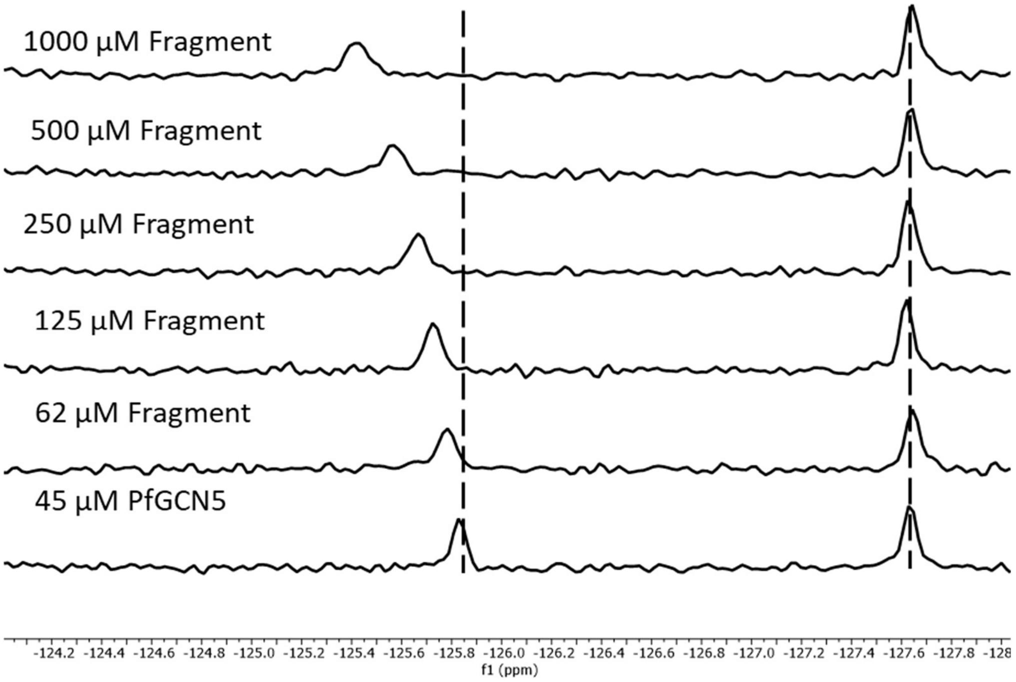

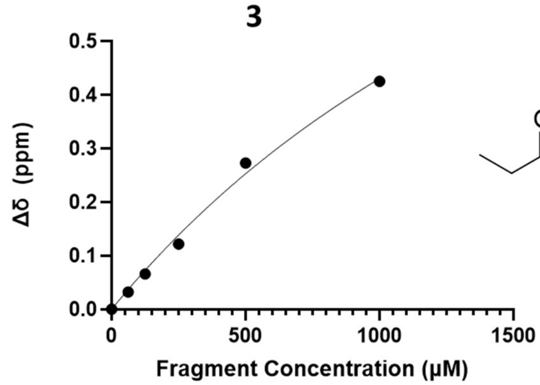

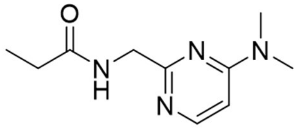

Titration of **4** with BPTF. No binding is observed.

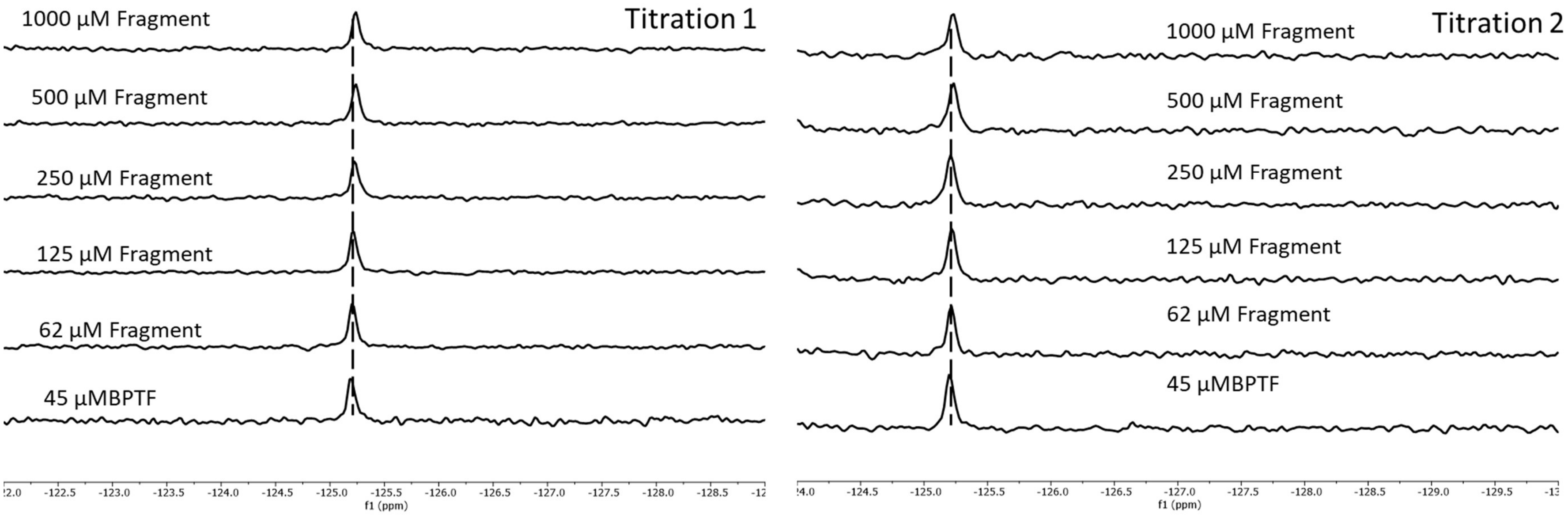

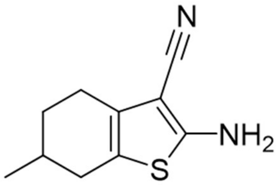

PrOF NMR titration of **5** with BPTF.

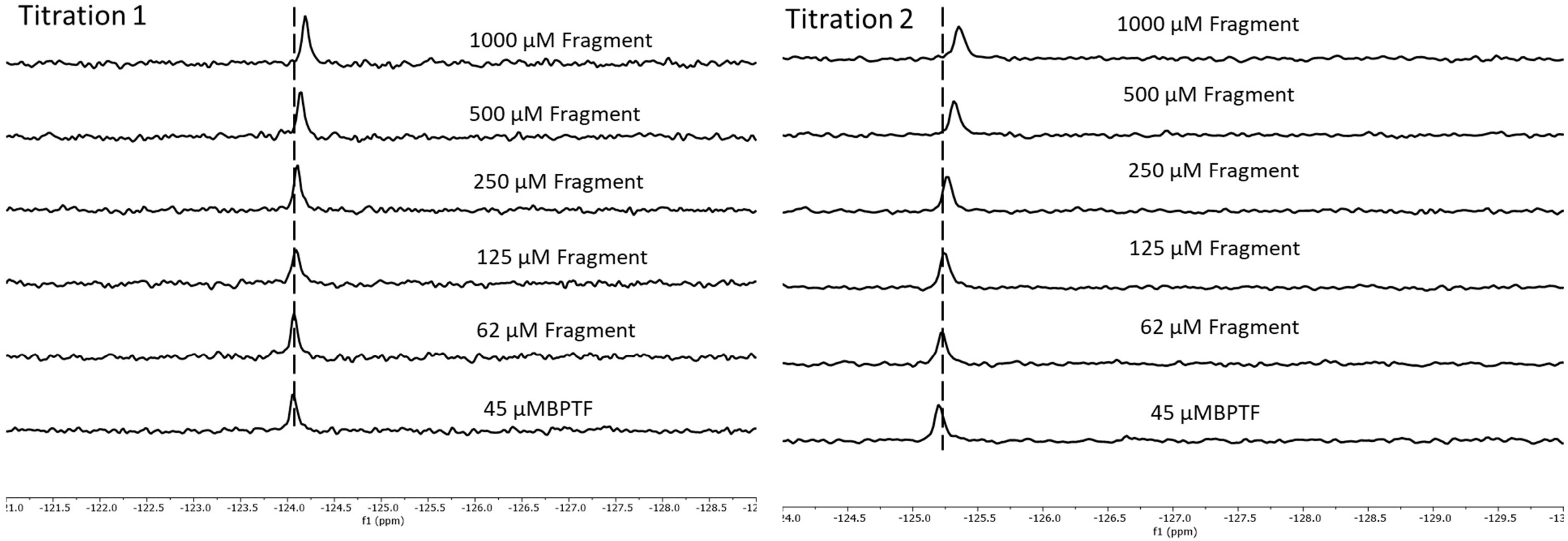

5

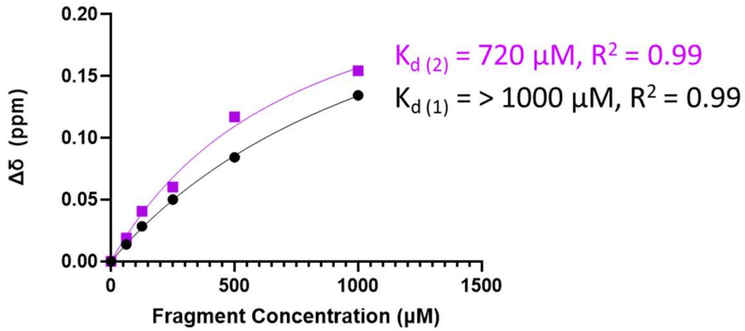

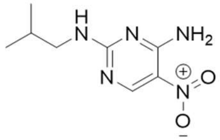

PrOF NMR titration of **6** with BPTF.

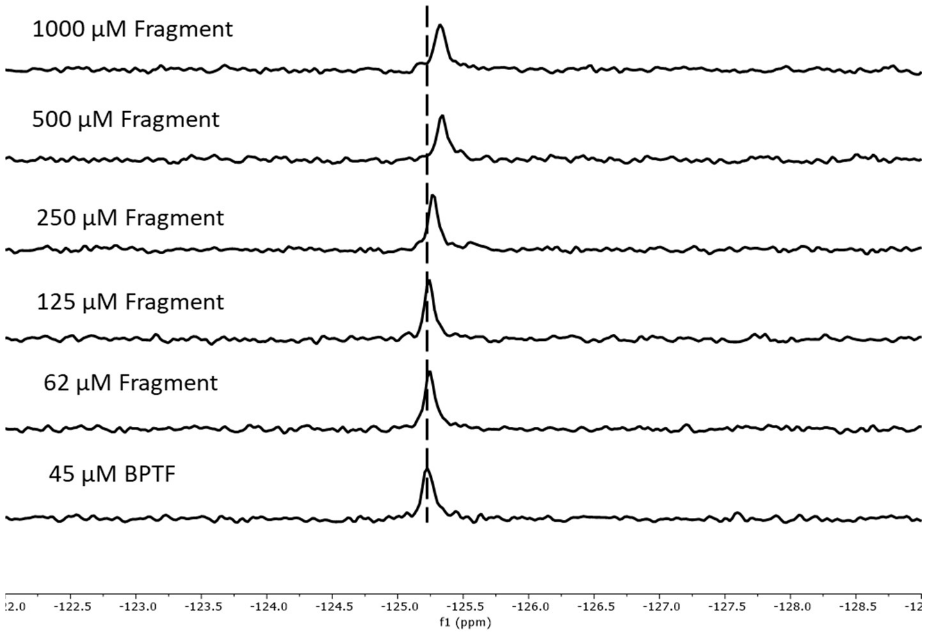

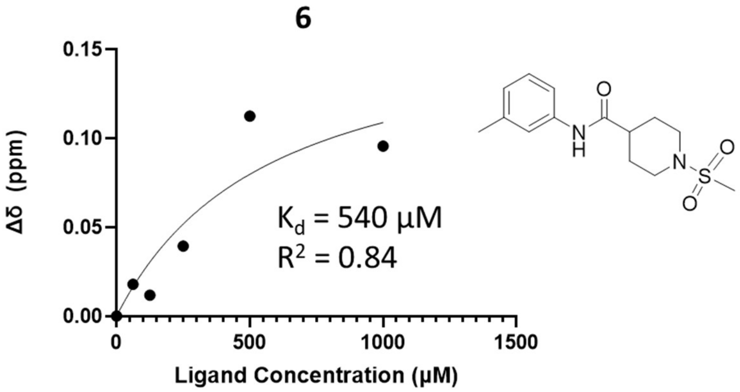

PrOF NMR titration of **7** with BPTF.

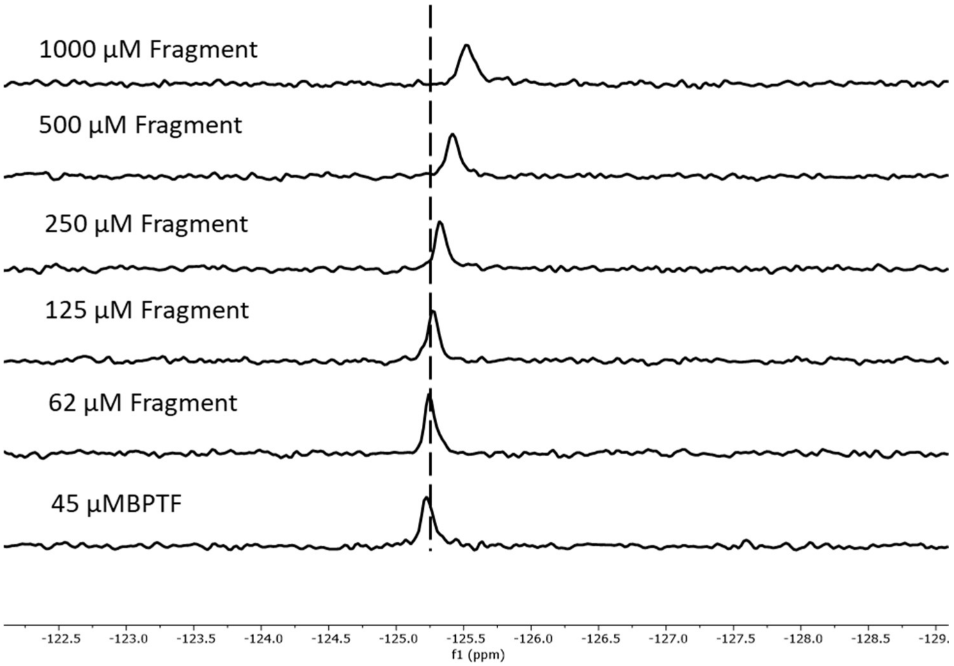

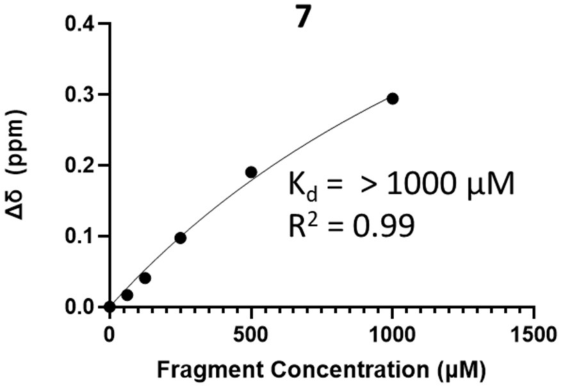

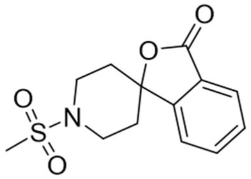

PrOF titration of **7** with *Pf*GCN5.

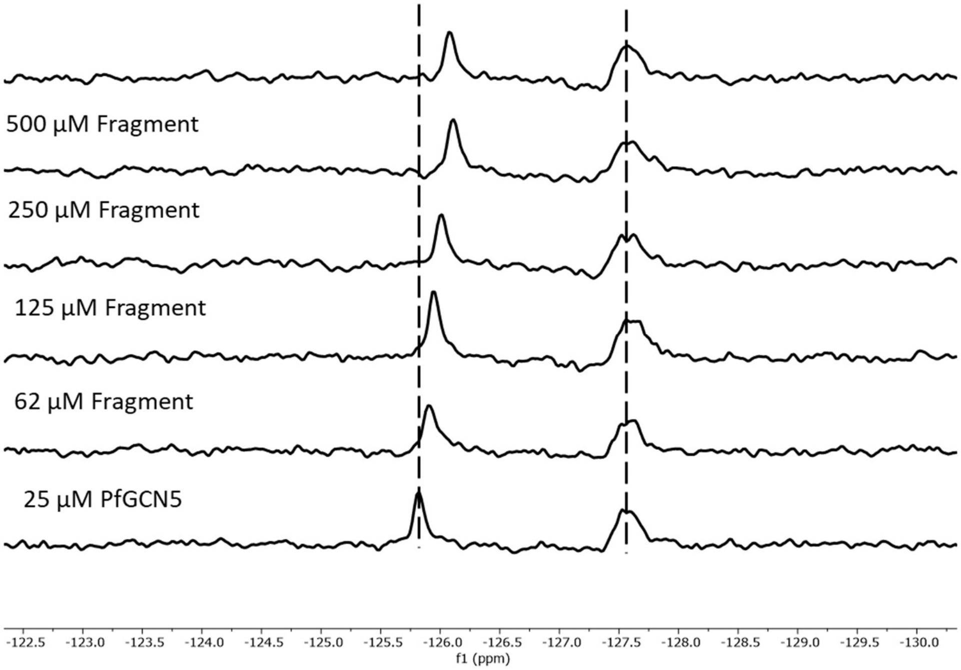

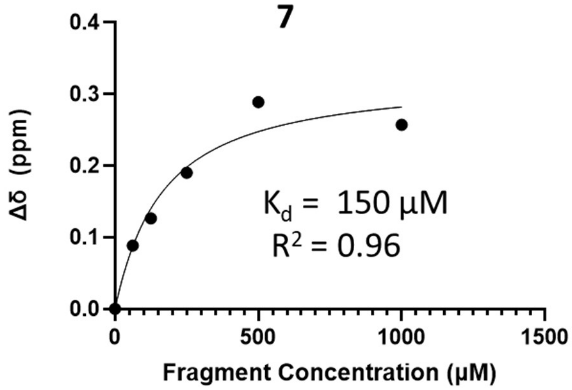

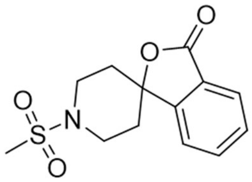

PrOF NMR titration of **8** with PfGCN5. Dose-dependent binding is observed but does not saturate.

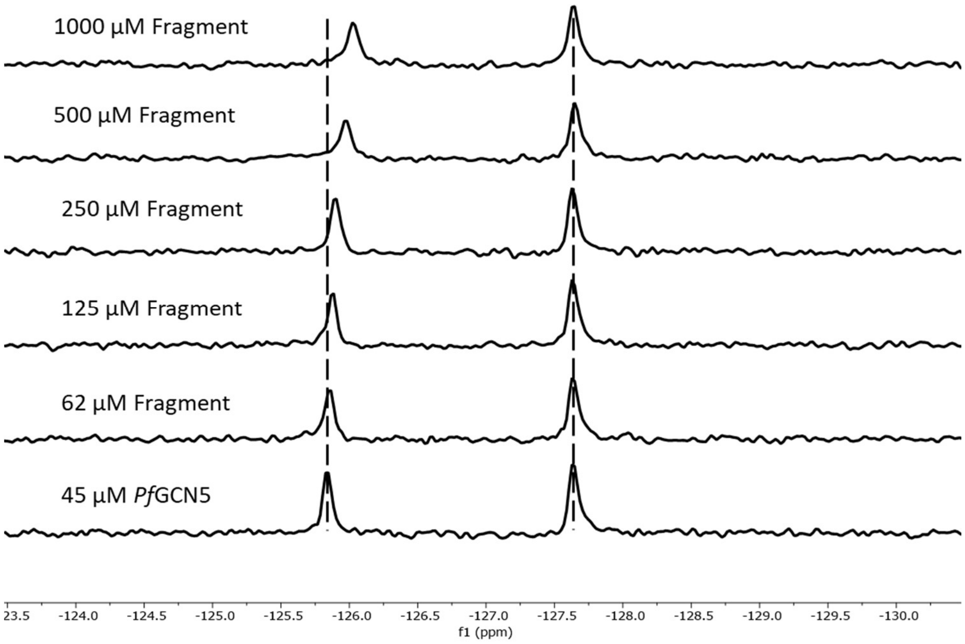

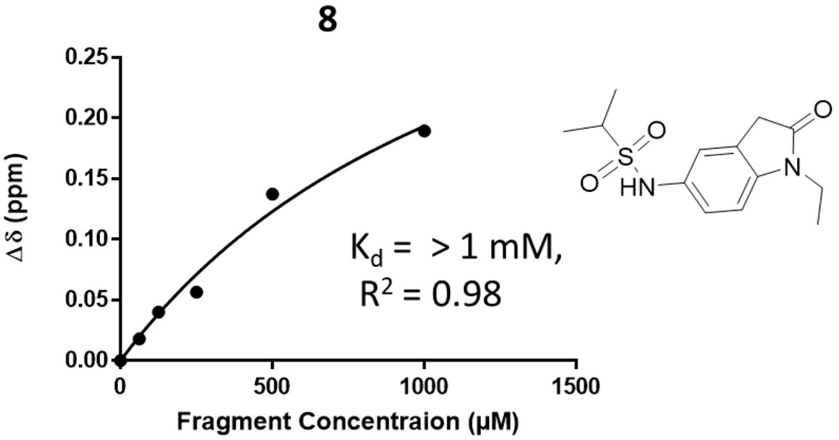

PrOF NMR titration of **8** with BPTF.

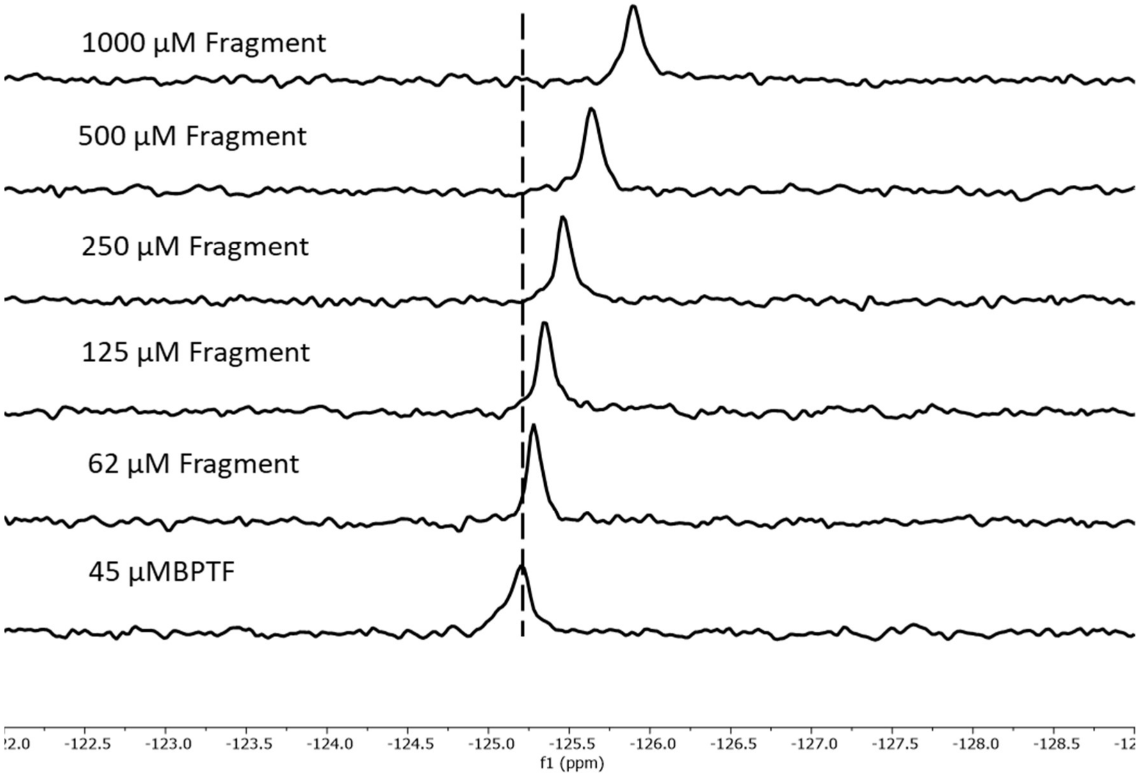

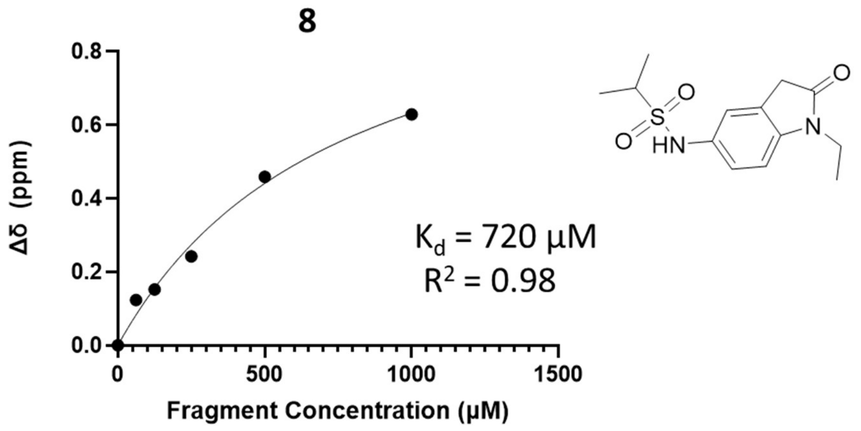

PrOF NMR titration of **9** with *Pf*GCN5. Movement in both W resoannces is observed. When fit the both resoances give similar a similar affinity.

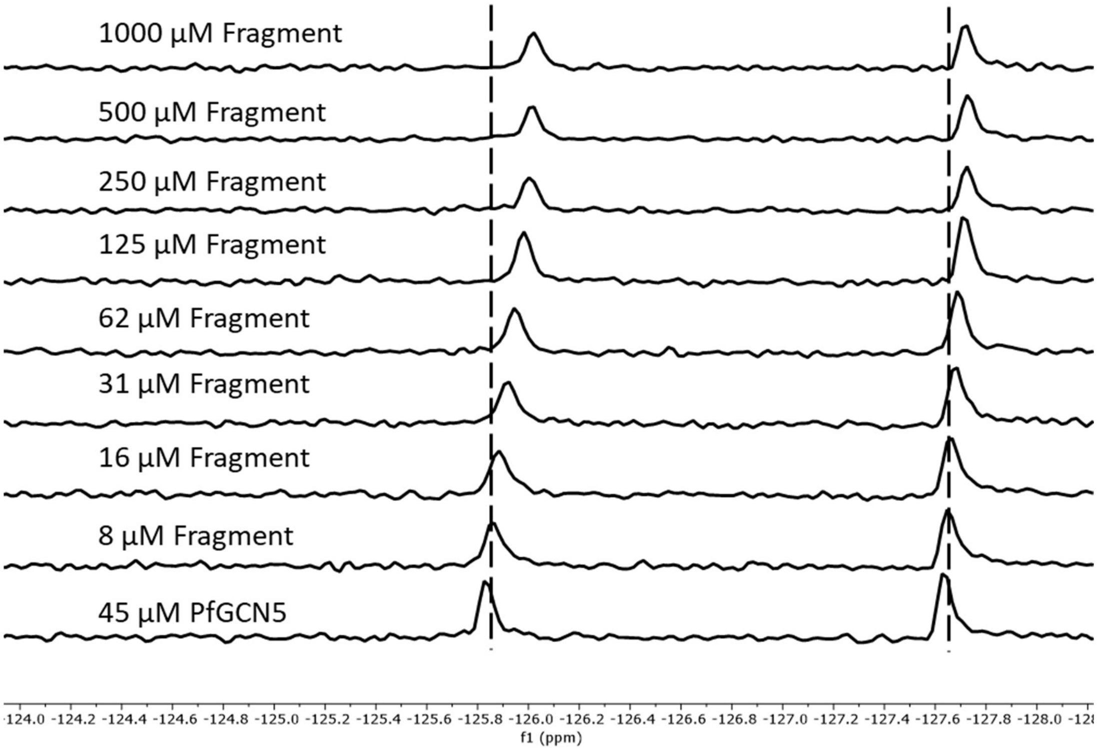

9

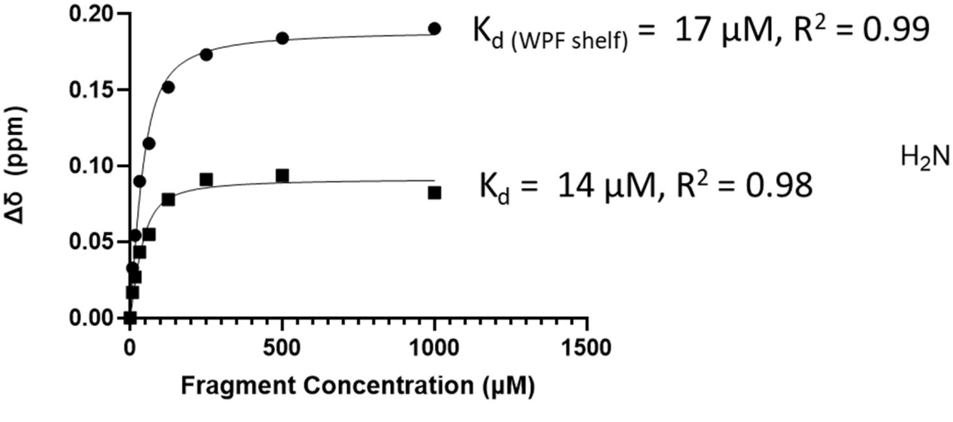

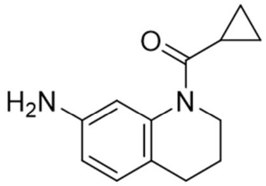

Titration of **H2A.Z II K7,13ac** with *Pf*GCN5**.**

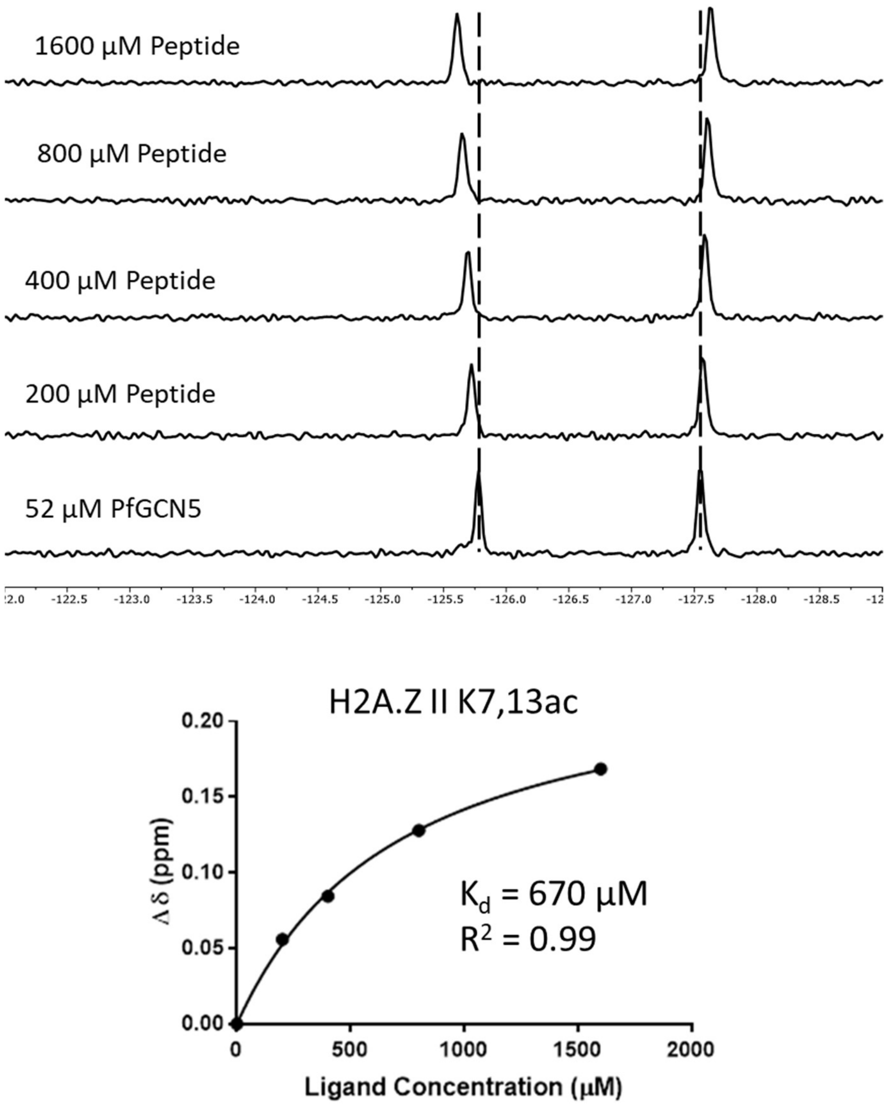

Peptide	Sequence	Calculated $[M+H]^+$	Observed $[M+H]^+$
H2A.Z II K7ac,K13ac	H_2N-YAGGKAGKacDSGKAKacAKAVSR-C(O)NH_2	2033.13	2032.64

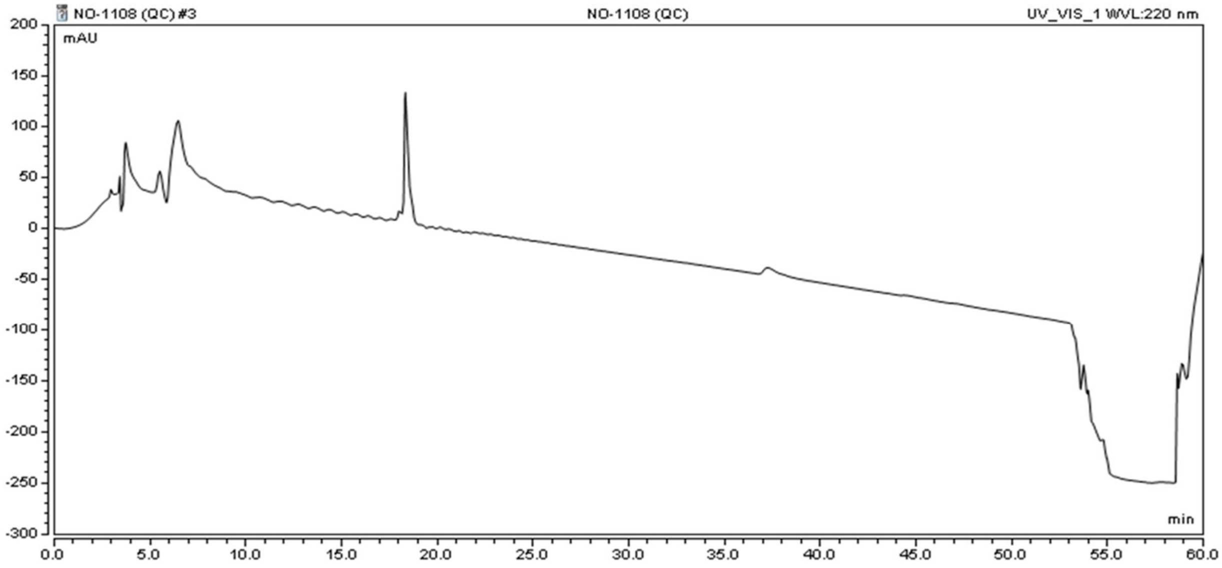

Figure S7. HPLC and MALDI mass spec data for H2A.ZII K7,13ac.

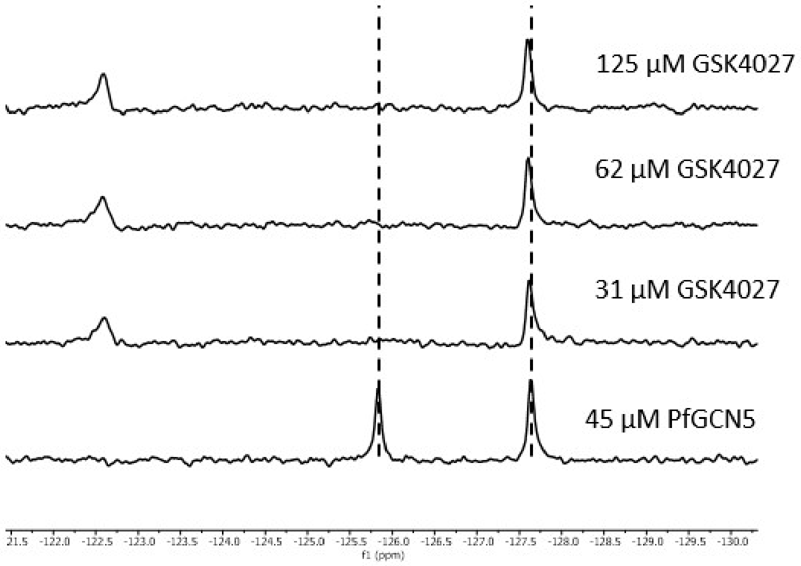

Figure 8. Titration of GSK4027 with *Pf*GCN5. Slow exchange is observed

A.

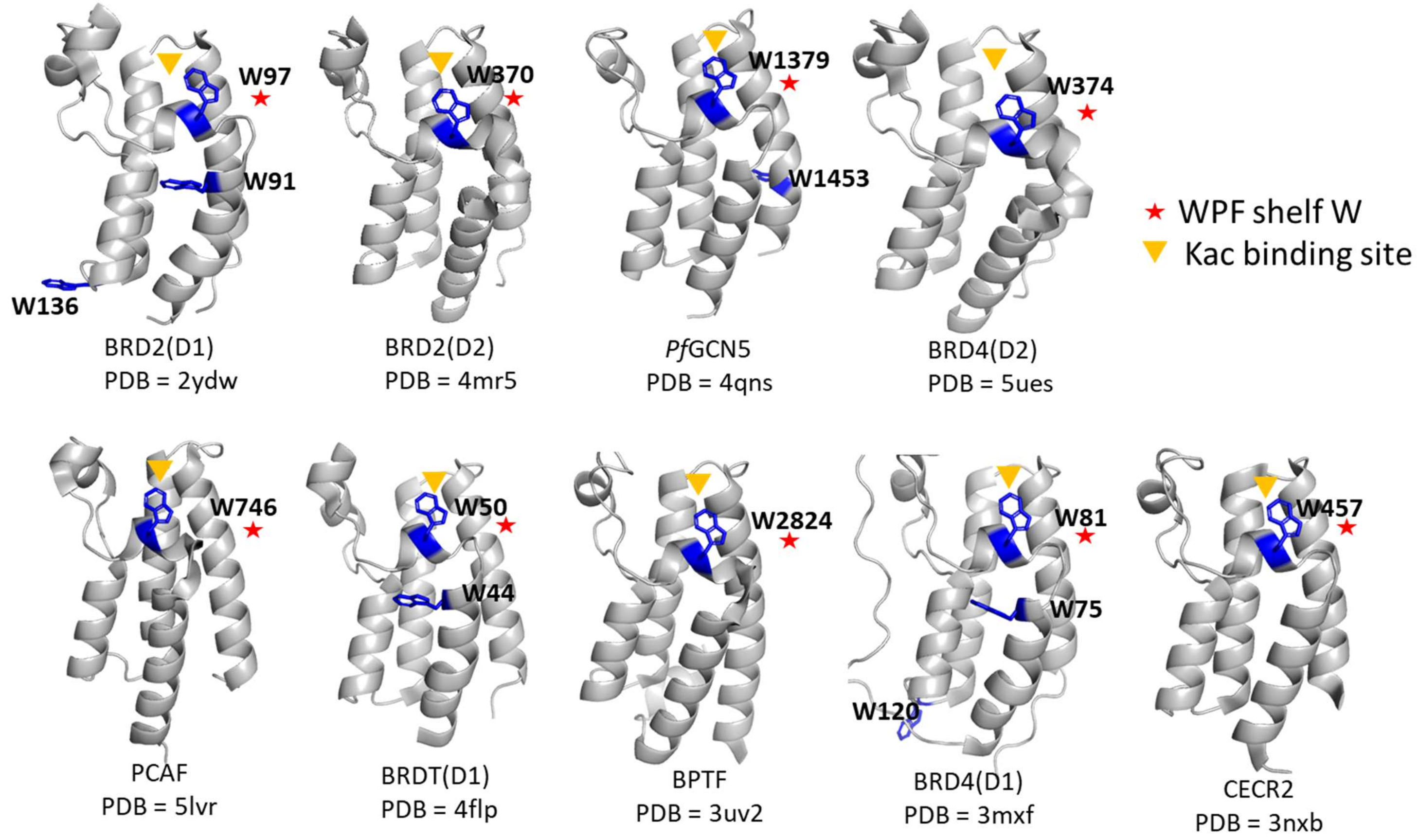

W97
W91
W136
BRD2(D1)
PDB = 2ydw
W370
BRD2(D2)
PDB = 4mr5
W1379
W1453
PfGCN5
PDB = 4qns
W374
BRD4(D2)
PDB = 5ues
WPF shelf W
Kac binding site
W746
PCAF
PDB = 5lvr
W50
W44
BRDT(D1)
PDB = 4flp
W2824
BPTF
PDB = 3uv2
W81
W75
W120
BRD4(D1)
PDB = 3mxf
W457
CECR2
PDB = 3nxb

B.

Protein	WPF W	Chemical Shift (ppm)
BRD2(D1)	91	-126.0992
BRD2(D2)	370	-126.7261
5FW PfGCN5	1379	-125.8523
5FW BRD4(D2)	374	-127.1832
5FW PCAF	746	-125.1181
5FW BRDT(D1)	50	-124.6154
5FW BPTF	2824	-125.235
5FW BRD4(D1)	81	-126.395
5FW CECR2	457	-126.1787

C.

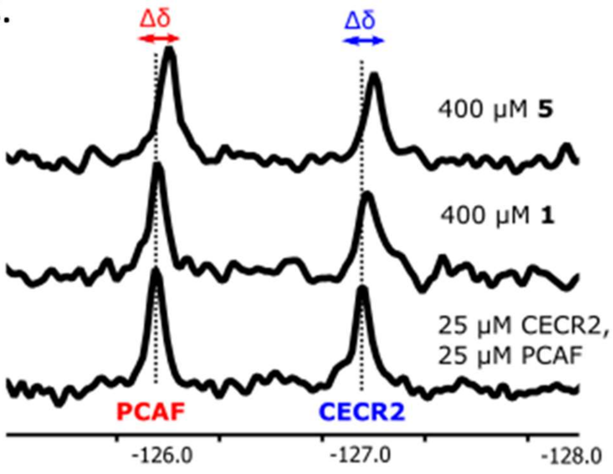

Figure S9. A) Locationg of W in various bromodomains. B) Chemical shifts of the 5FW resonance of WPF shelf W for various bromodomains. C) Example dual protien PrOF NMR assay with fragments 1 and 5 with two bromdomain family IV proteins CECR2 and PCAF.

Table S5. Competition ^{1}H CPMG NMR of **9**.

	**Resonances 9**		**Kac resonances of H2A.Z II K7,13ac**
	**6.98 ppm**	**2.6884 ppm**	**1.99 ppm**
% drop *Pf*GCN5	71	72	62
% recovery with 10 uM GSK	30	14	99
% recovery with 20 uM GSK	29	26	95
% recovery with 35 uM GSK	35	37	107
% recovery with 50 uM GSK	57	34	
% recovery with 100 uM GSK	54	52	
% drop PfGCN5	75	83	
% recovery with 10 uM L-Moses	13	17	
% recovery with 20 uM L-Moses	60	61	
% recovery with 35 uM L-Moses	51	57	
% recovery with 50 uM L-Moses	40	44	
% recovery with 100 uM L-Moses	40	58	
% drop BPTF	68	46	
% recovery with 10 uM TP238	88	86	
% recovery with 20 uM TP238	87	76	

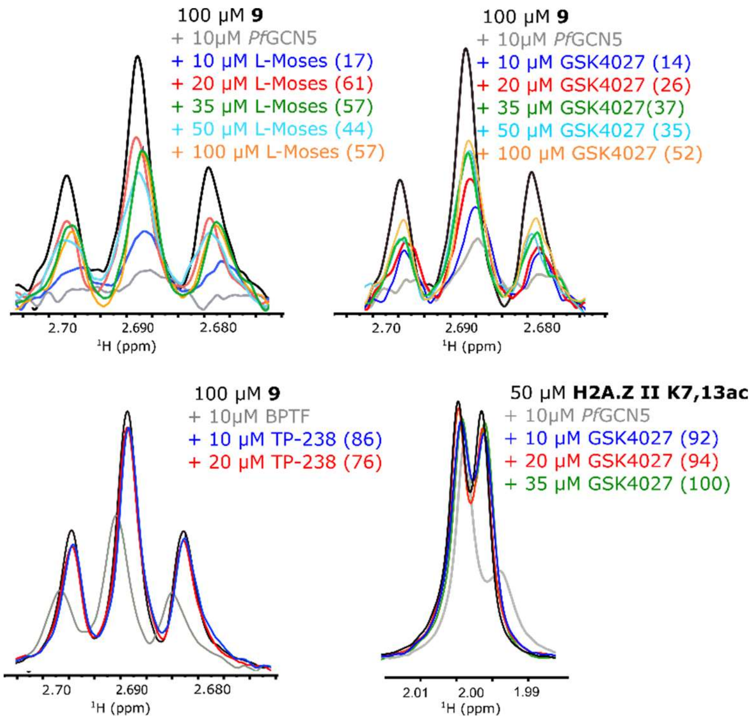

Figure S10. ^{1}H CPMG NMR comptetiton experiments overaly of spectra. Number in paranteses is the % recovery of the resonance**.**

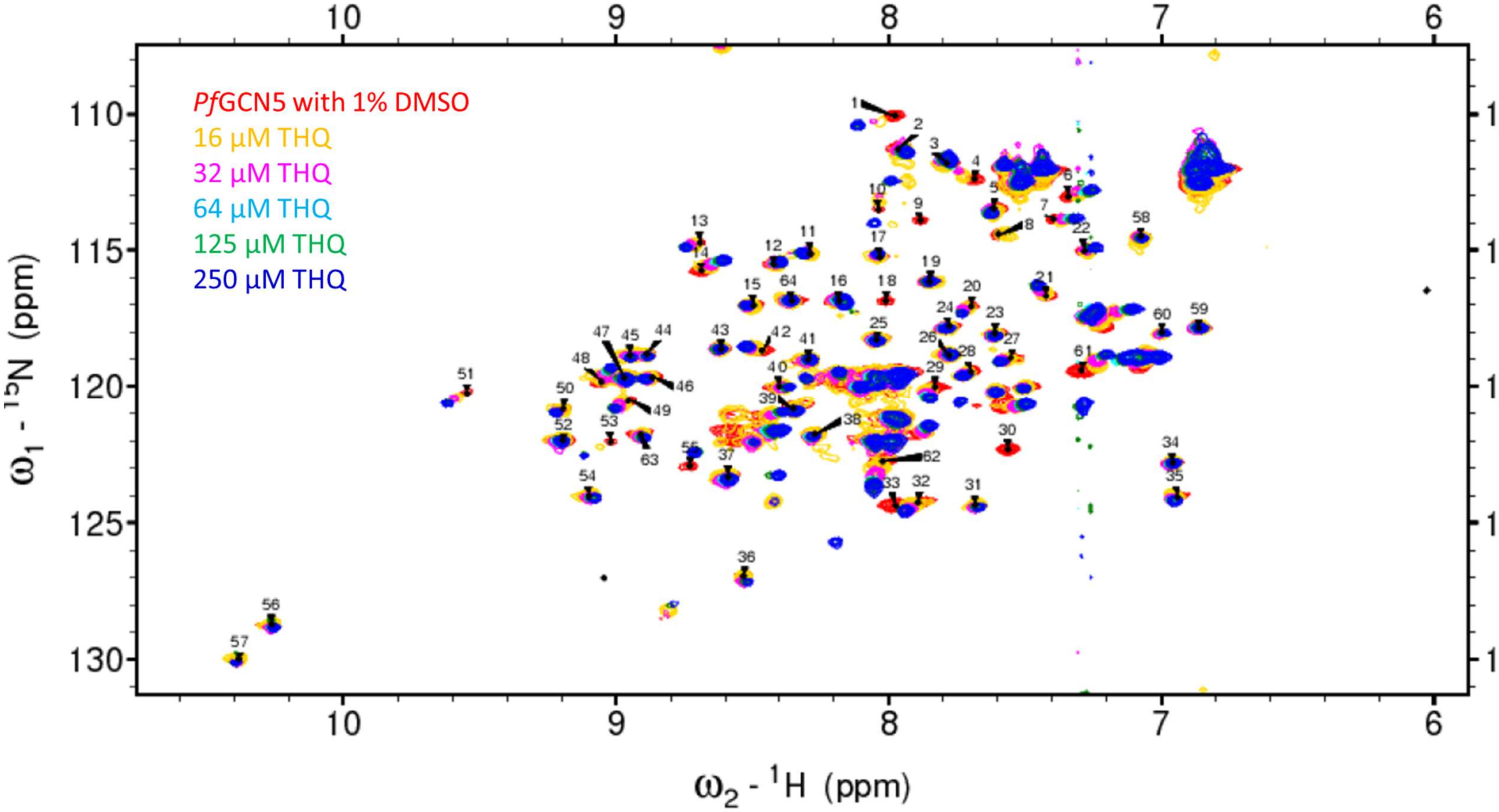

PfGCN5 with 1% DMSO
16 µM THQ
32 µM THQ
64 µM THQ
125 µM THQ
250 µM THQ
ω1 - 15N (ppm)
ω2 - 1H (ppm)

B.

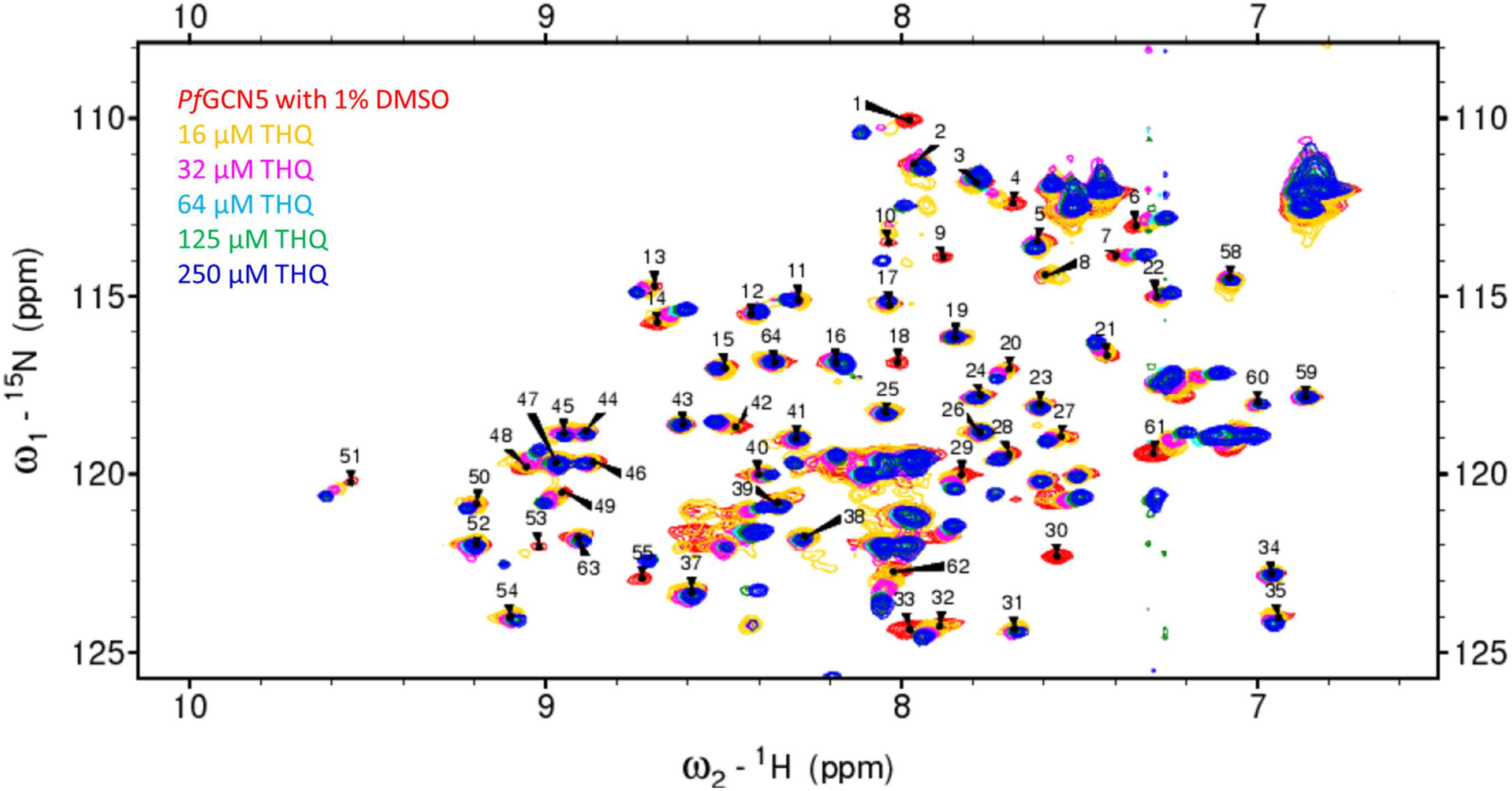

PfGCN5 with 1% DMSO
16 µM THQ
32 µM THQ
64 µM THQ
125 µM THQ
250 µM THQ
ω2 - 1H (ppm)
ω1 - 15N (ppm)

C.

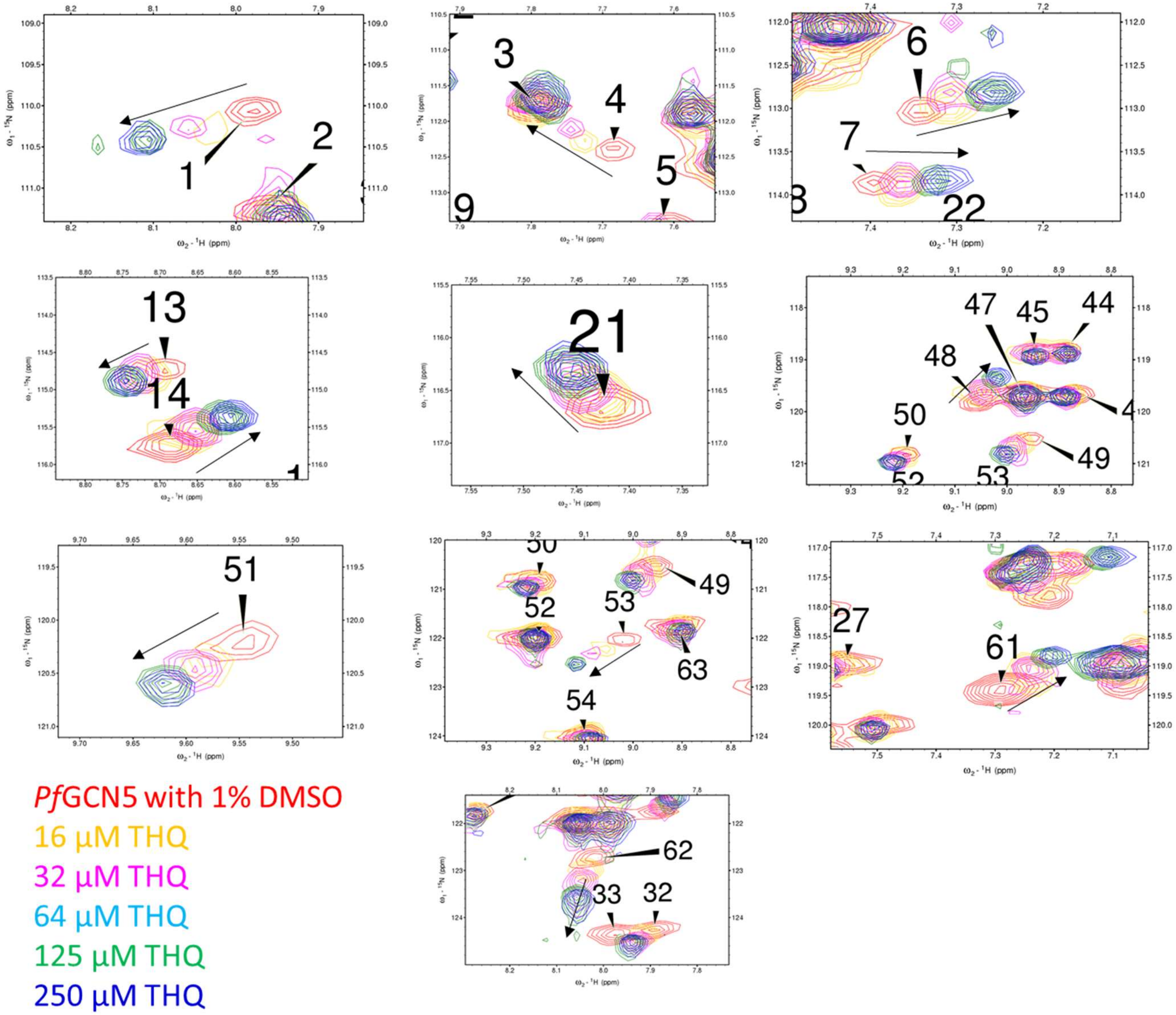

PfGCN5 with 1% DMSO
16 μM THQ
32 μM THQ
64 μM THQ
125 μM THQ
250 μM THQ

Figure S11. ^{1}H -^{15}N HSQC titration of **9** with *Pf*GCN5. A) Full spectrum B) Zoomed in on the congested portion of the spectrum C) Zoomed in on various portions of the full spectrum. C) Inlays of cross peaks used to calculate the K_d of **9**.

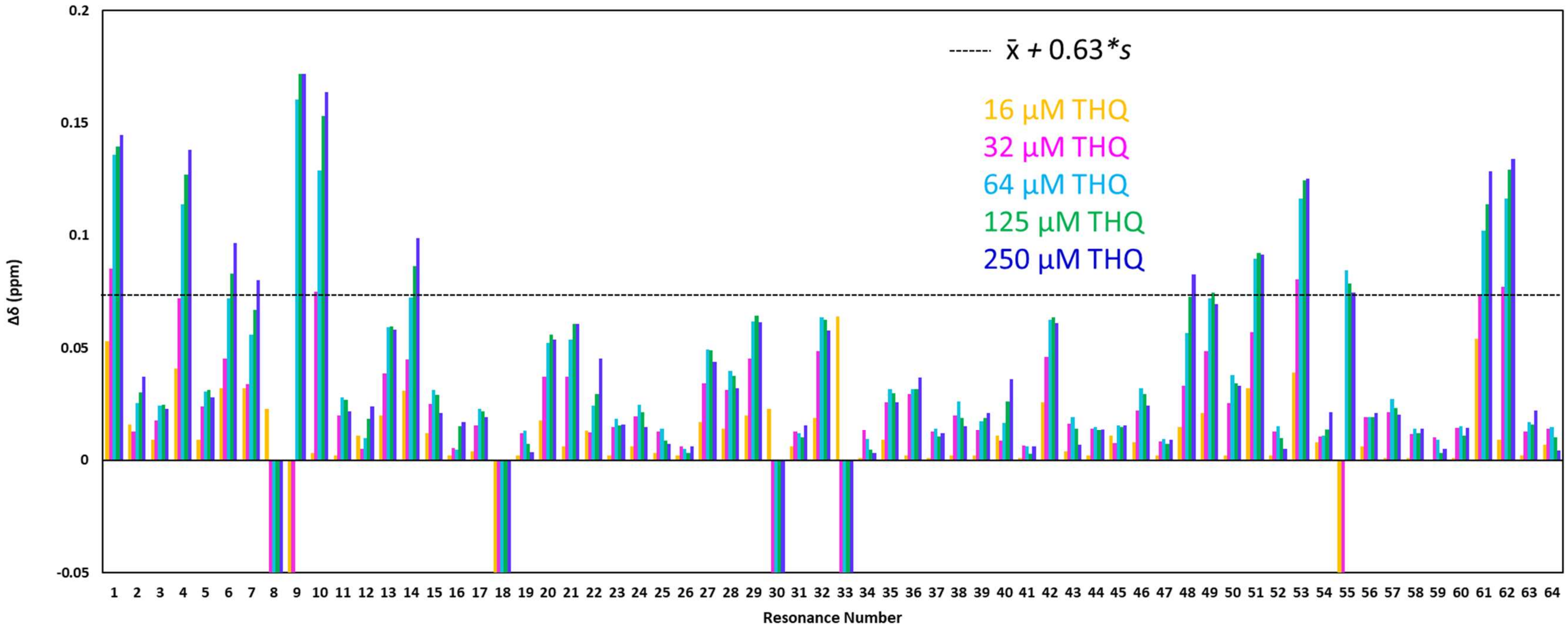

Figure S12. Δδ for each residue for the ^{1}H -^{15}N HSQC titration of **9** with *Pf*GCN5. The cross peaks at -0.05 ppm are cross peaks that disappeared. The dotted line represents the average plus 60% of one standard deviation from the mean at the highest concentration of **9**.

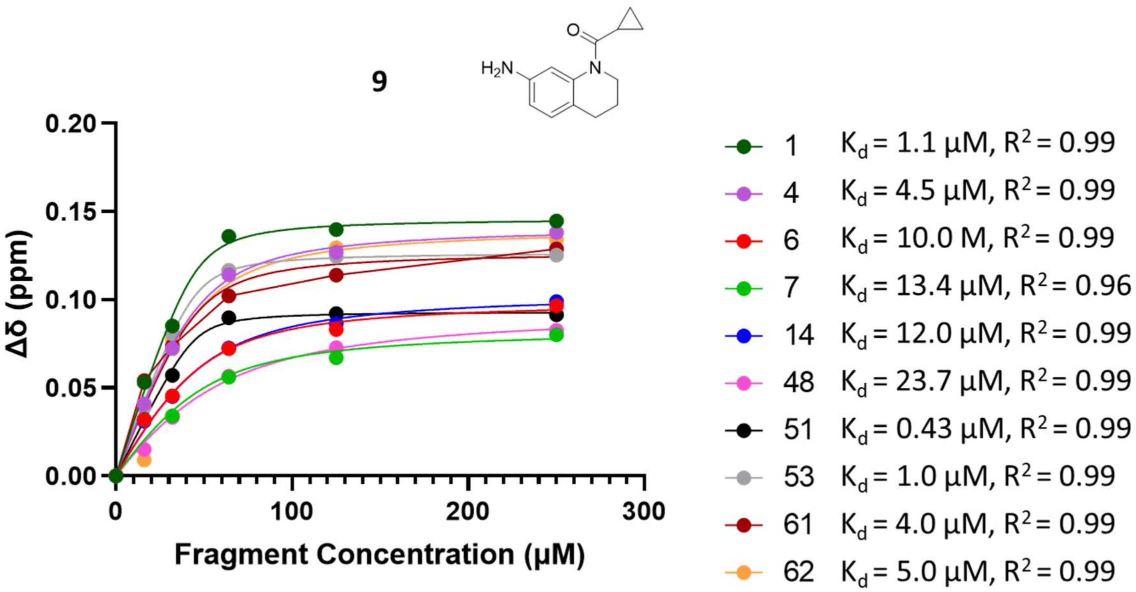

Average K_d = 7.5 μM, *s* = 7.0 μM, LE = 0.47

Average K_d without *51* = 8.3 μM, *s* = 7.0 μM, LE = 0.46

Figure S13. Titration isotherms for the ^{1}H -^{15}N HSQC titration of **9** with 50 μM ^{15}N labeled *Pf*GCN5. Residues that moved linearly were used to determine the K_d. Resonances *1, 4, 6, 51,* and *53* move in a linear fashion to a significant extent in the titration with 9 but not in the titration with the H2A.Z II K7,13ac peptide. The low Kd based on total protein concentration is likely to contribute to error in the affinity calculation.

A.

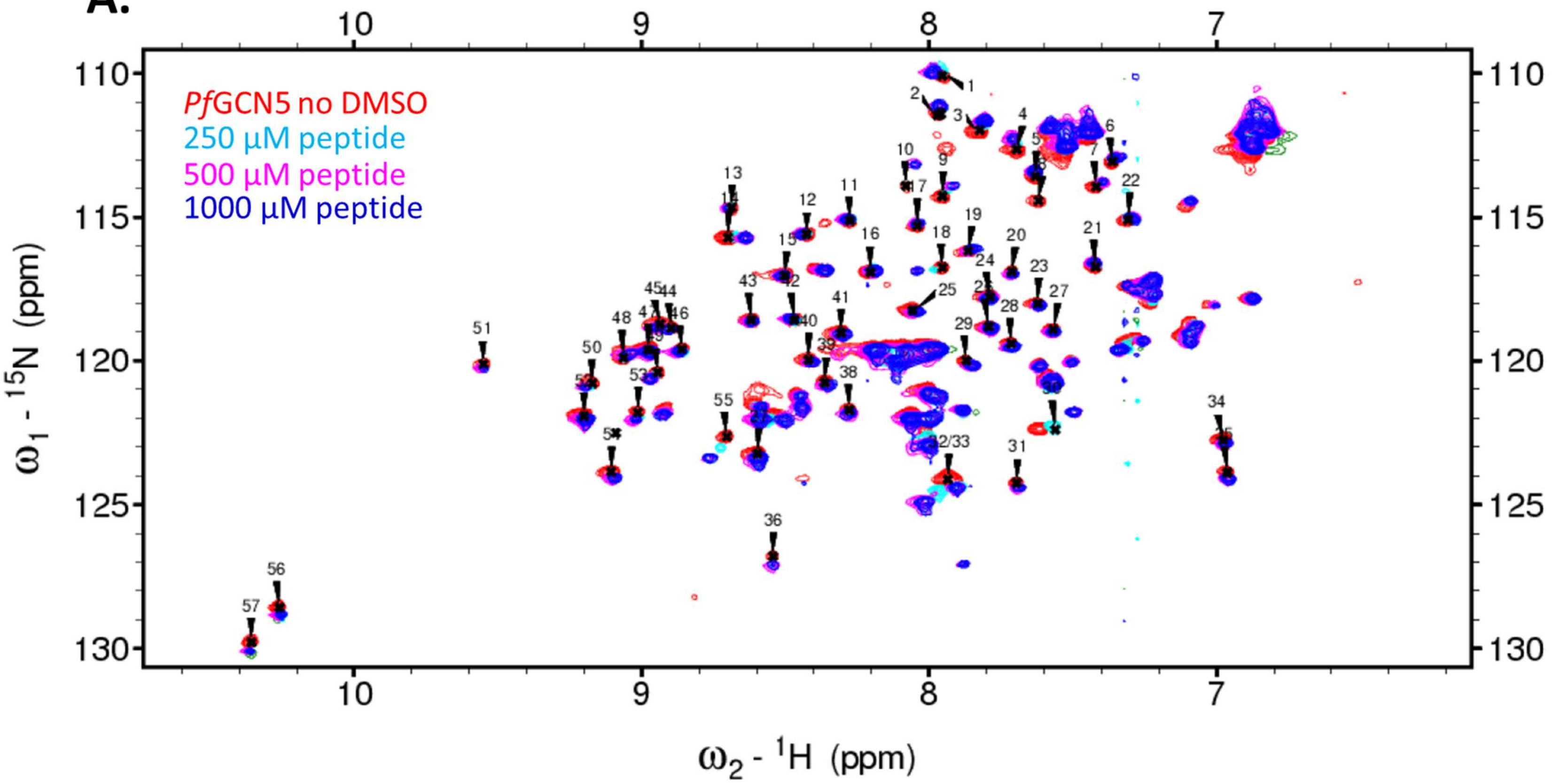

*Pf*GCN5 no DMSO
250 µM peptide
500 µM peptide
1000 µM peptide
ω1 - 15N (ppm)
ω2 - 1H (ppm)

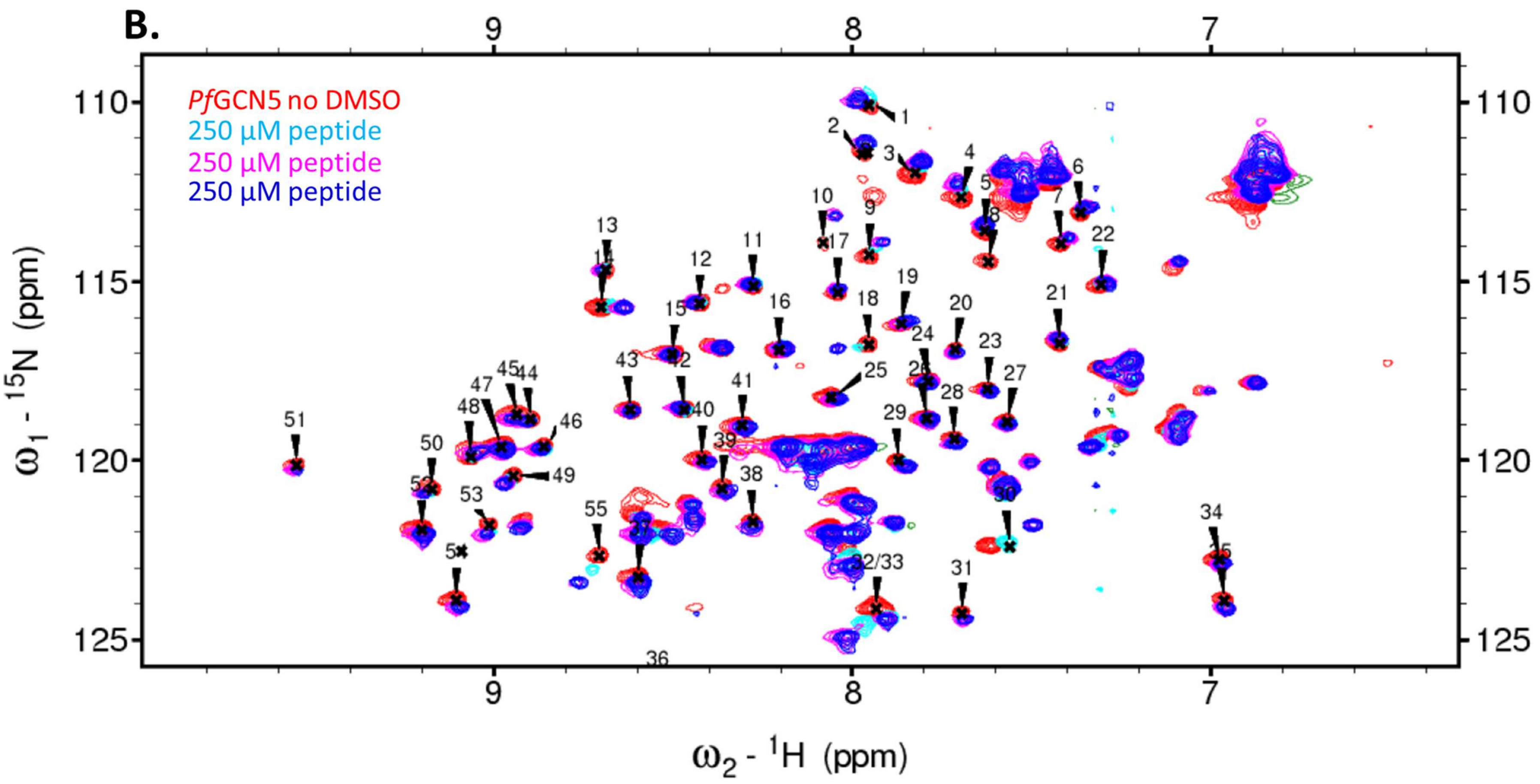

Figure S14. ^{1}H -^{15}N HSQC titration of H2A.Z II K7,13ac with *Pf*GCN5. A) Full spectrum B) Zoomed in on the congested portion of the spectrum C) Zoomed in on various portions of the full spectrum.

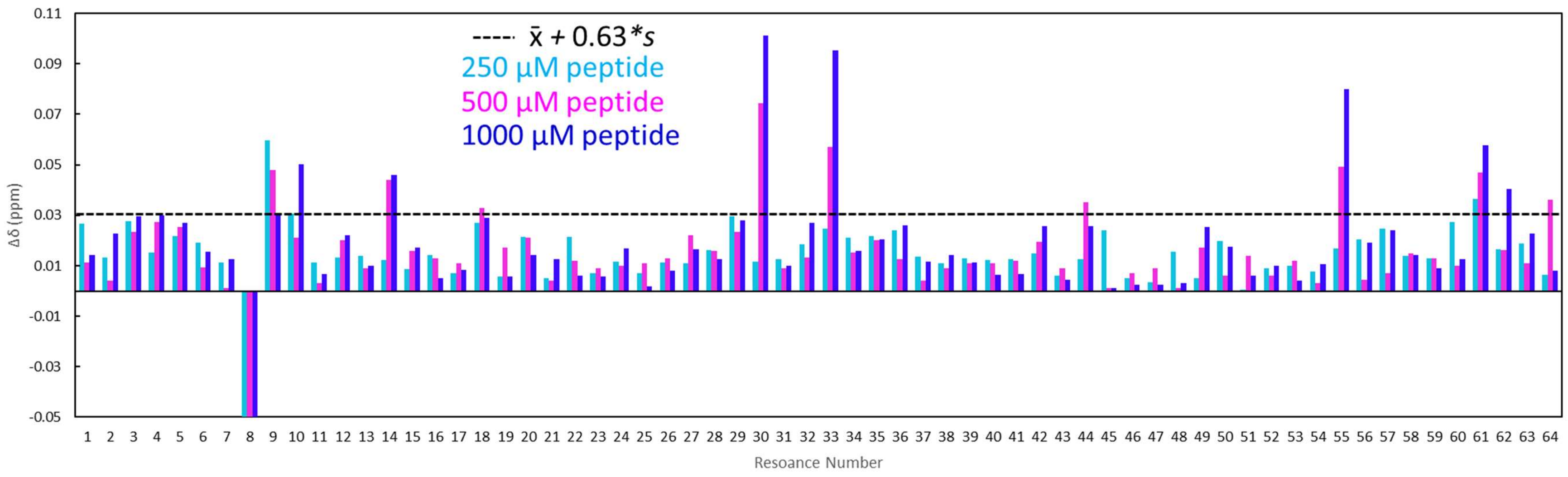

Figure S15. Δδ for each residue for the ^{1}H -^{15}N HSQC titration of H2A.Z II K7,13ac with *Pf*GCN5. The cross peaks at -0.05 ppm are cross peaks that disappeared. The dotted line represents the average plus 60% of one standard deviation from the mean at the highest concentration of peptide.

A.

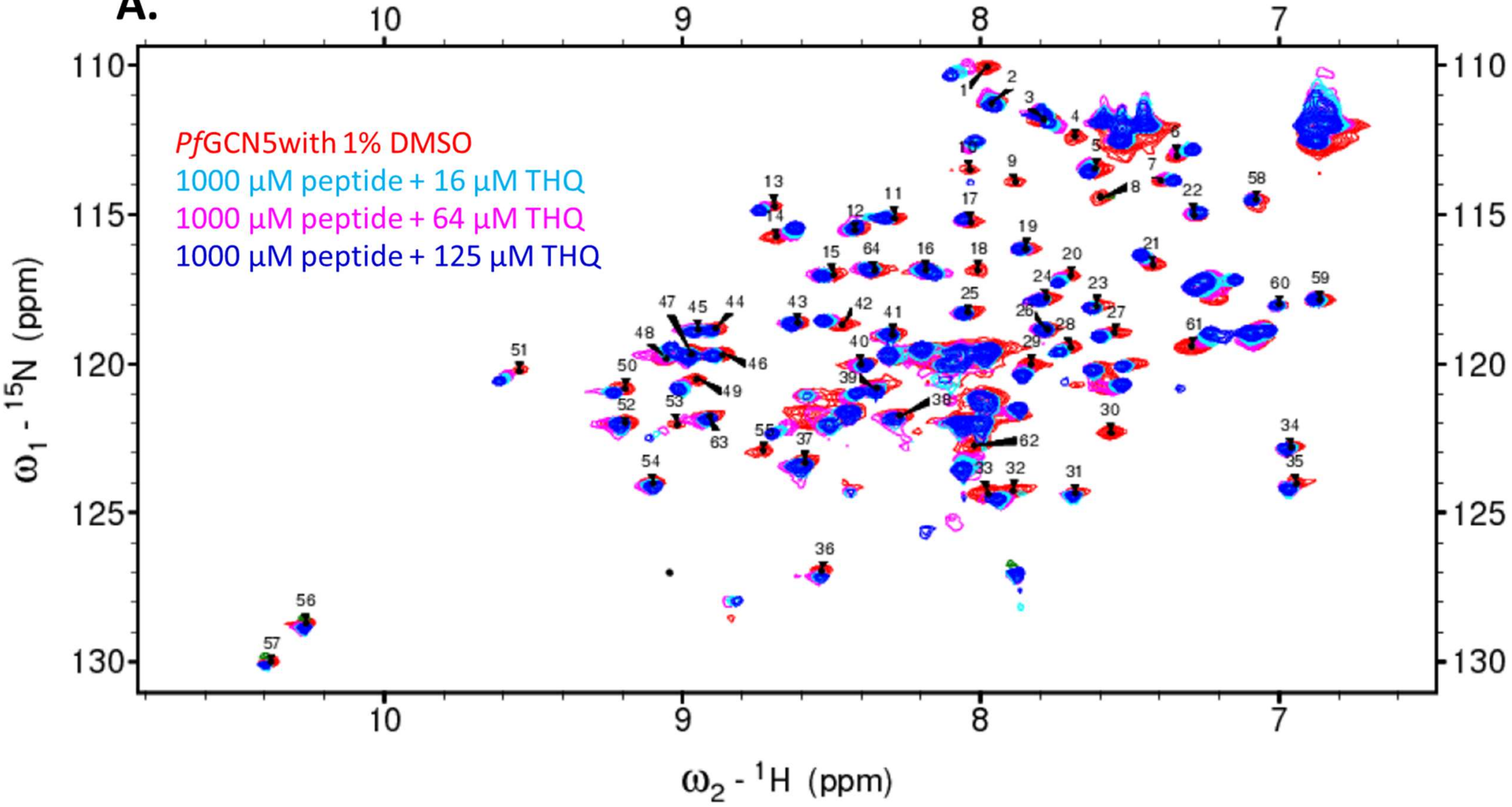
PfGCN5with 1% DMSO
1000 µM peptide + 16 µM THQ
1000 µM peptide + 64 µM THQ
1000 µM peptide + 125 µM THQ
ω1 - 15N (ppm)
ω2 - 1H (ppm)

B.

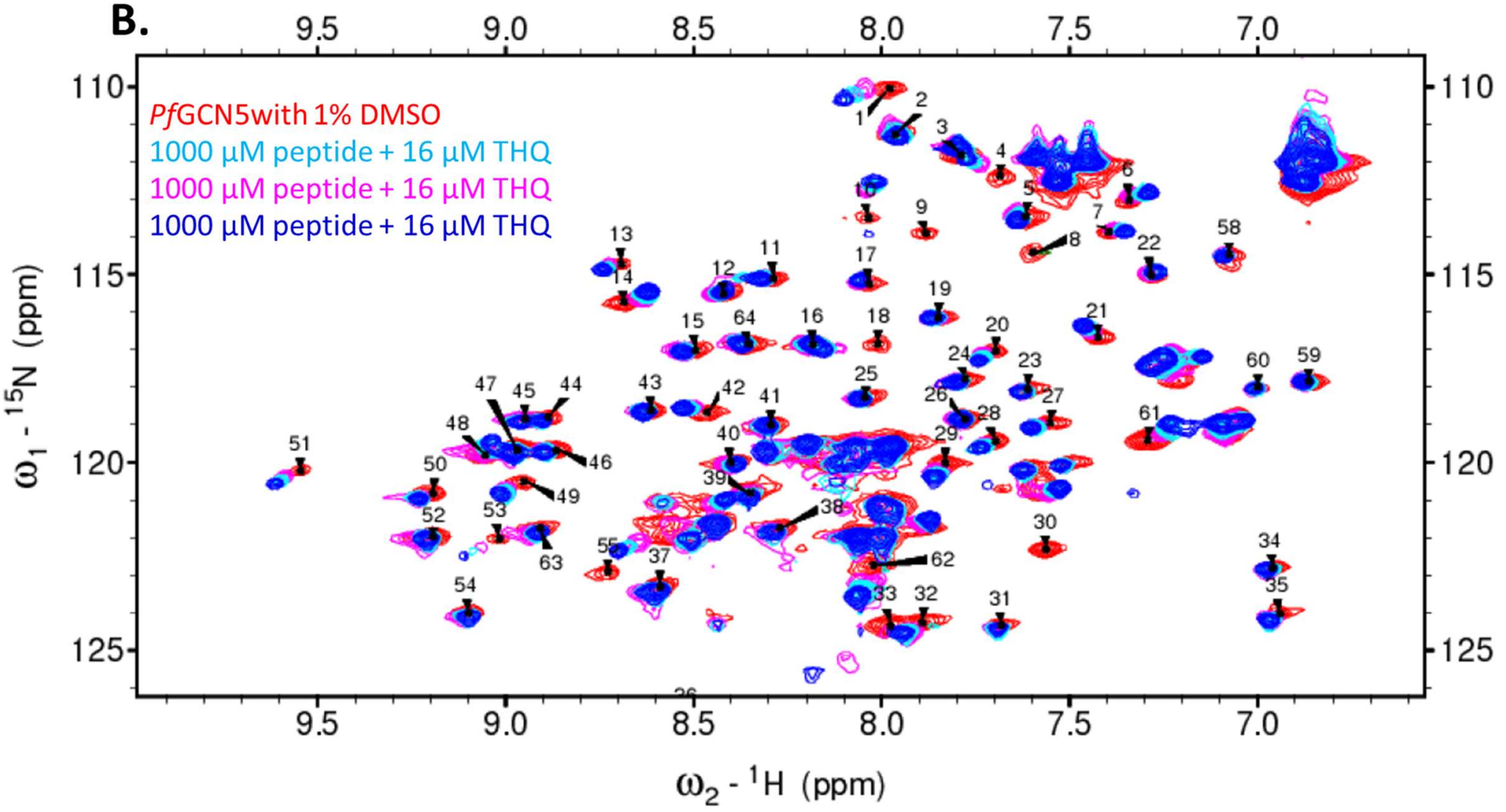

Figure S16. ^{1}H -^{15}N HSQC titration of **9** with a saturating concentration of H2A.Z II K7,13ac with *Pf*GCN5. A) Full spectrum B) Zoomed in on the congested portion of the spectrum.

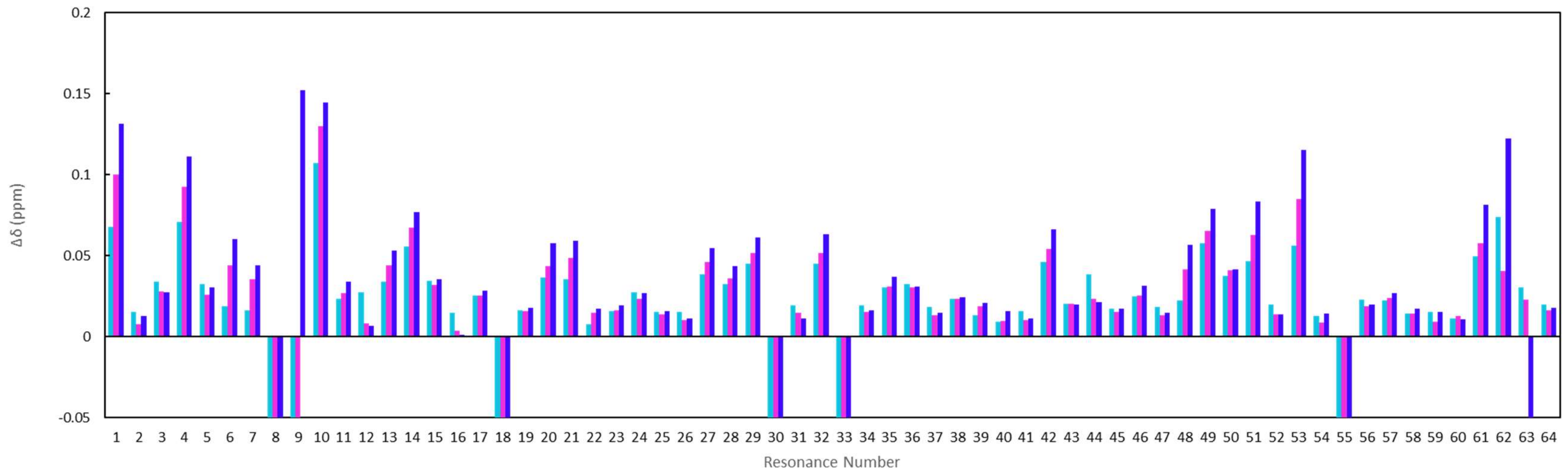

Figure S17. Δδ for each residue for the ^{1}H -^{15}N HSQC titration of **9** with a saturating concentration of H2A.Z II K7,13ac with *Pf*GCN5. The cross peaks at -0.05 ppm are cross peaks that disappeared. The dotted line represents the average plus 60% of one standard deviation from the mean at the highest concentration of **9**.

A.

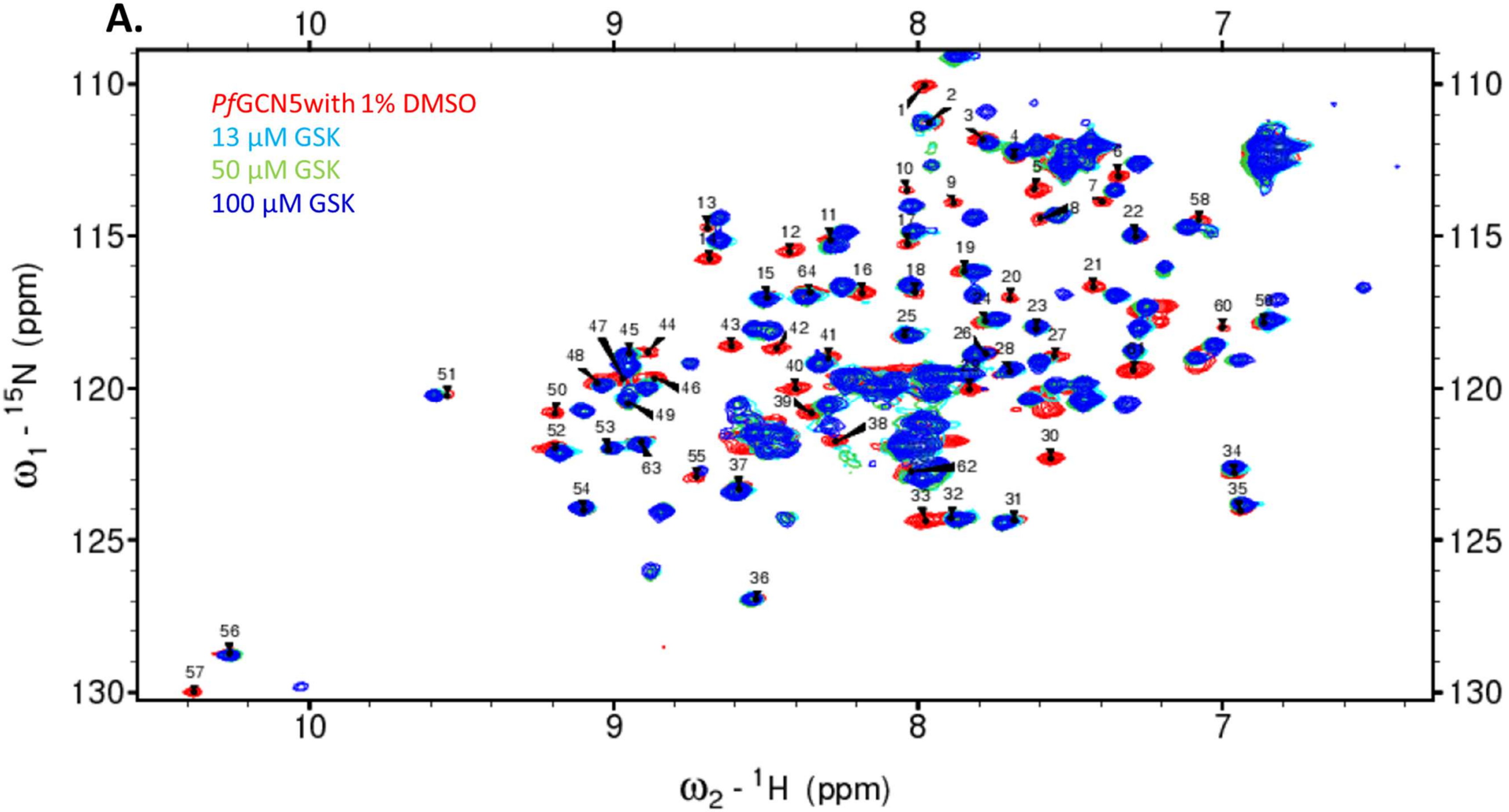

PfGCN5with 1% DMSO
13 μM GSK
50 μM GSK
100 μM GSK
ω1 - 15N (ppm)
ω2 - 1H (ppm)

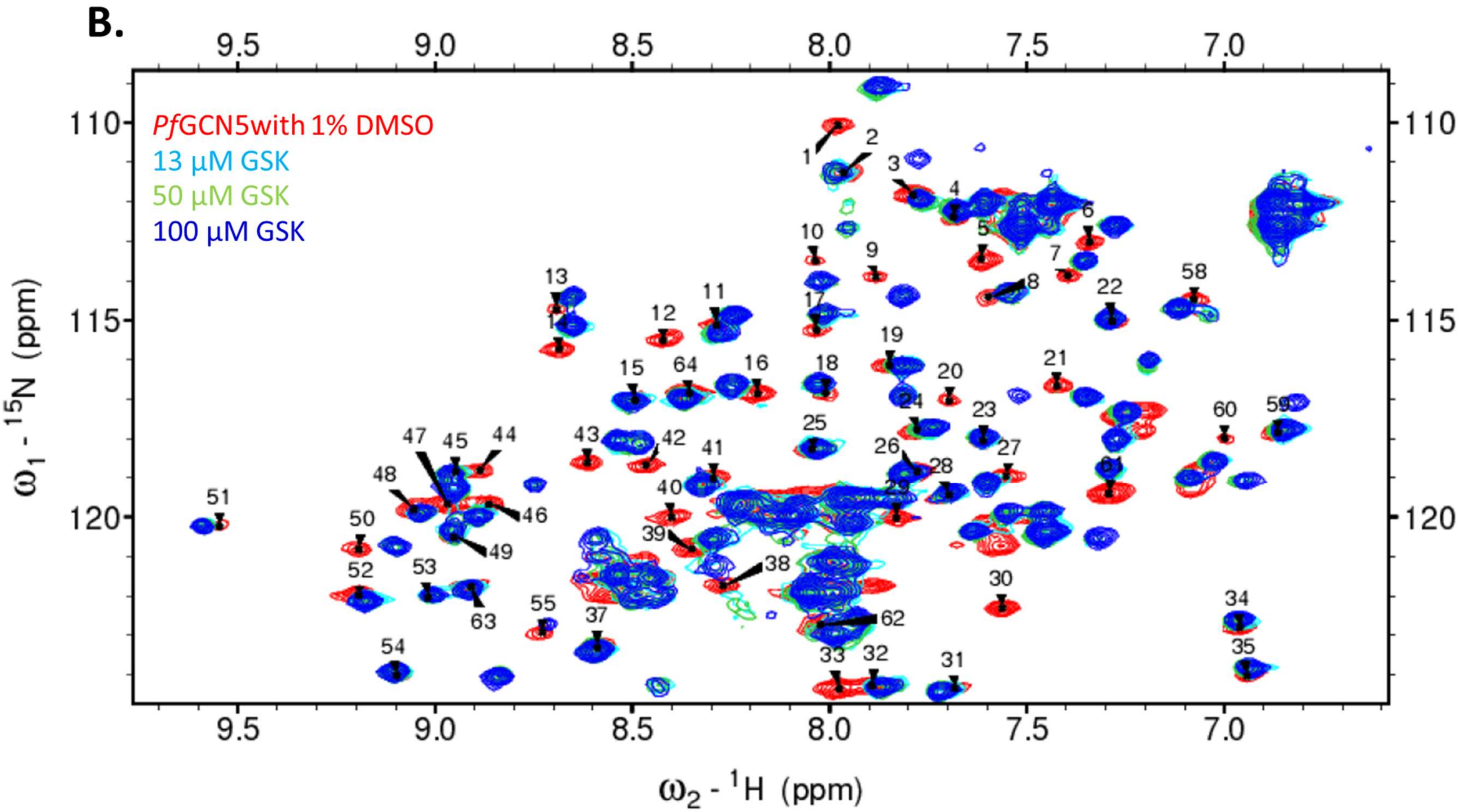

Figure S18. ^{1}H -^{15}N HSQC titration of GSK4207 with *Pf*GCN5. A) Full spectrum B) Zoomed in on the congested portion of the spectrum.

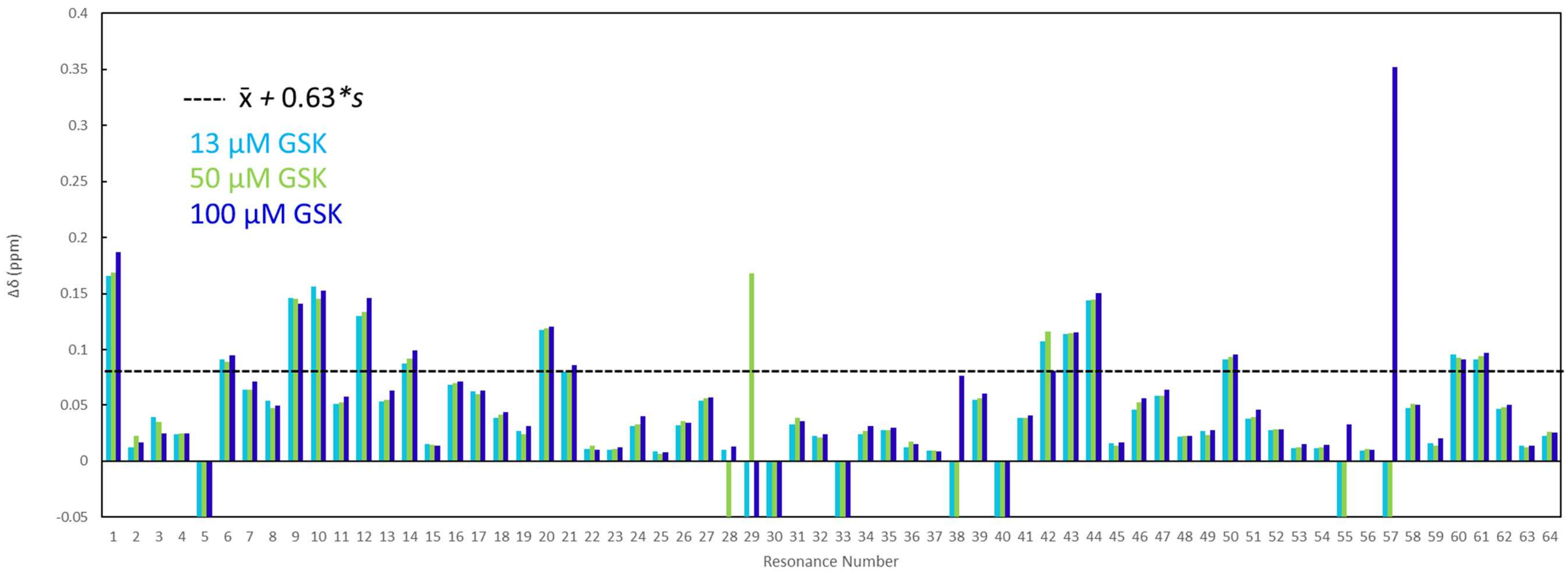

Figure S19. Δδ for each residue for the ^{1}H -^{15}N HSQC titration of GSK4027 with *Pf*GCN5. The cross peaks at -0.05 ppm are cross peaks that disappeared. The dotted line represents the average plus 60% of one standard deviation from the mean at the highest concentration of GSK4027.

The FTMap[2-4] server was used to identify hotspots on the PfGCN5 bromodomain (PDB ID = 4QNS, chain A). Fifteen Consensus clusters were identified (Fig S20). Only one cluster with more than 4 probes was identified near the distal W (W1453); this cluster contained 12 probe molecules and was the third highest occupancy cluster. The two highest occupancy clusters were found in the Kac binding site.

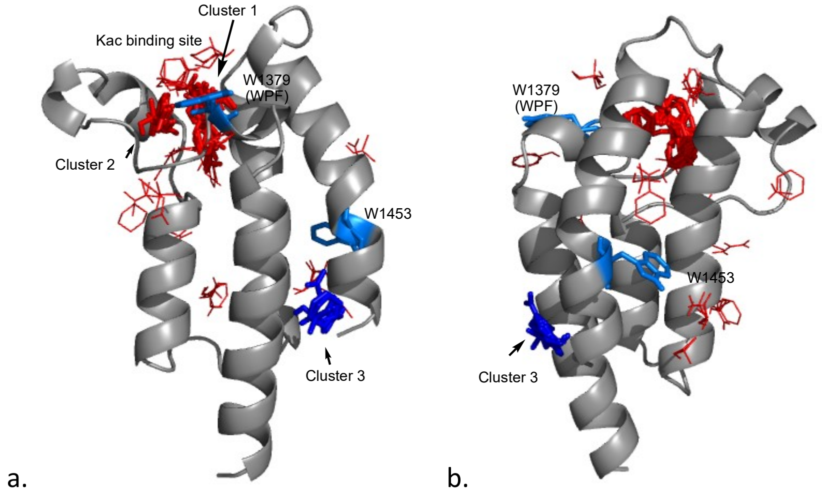

Figure S20. Hotspots on PfGCN5 bromodomain (protein in gray, tryptophan residues in light blue) as identified by FTMap. Consensus Clusters are shown in red lines, and the two highest priority clusters are highlighted in red sticks while the third highest priority cluster is highlighted in dark blue. **a.** view with the WPF shelf in front, distal W in back; **b.** view with WPF shelf in back, distal W in front.

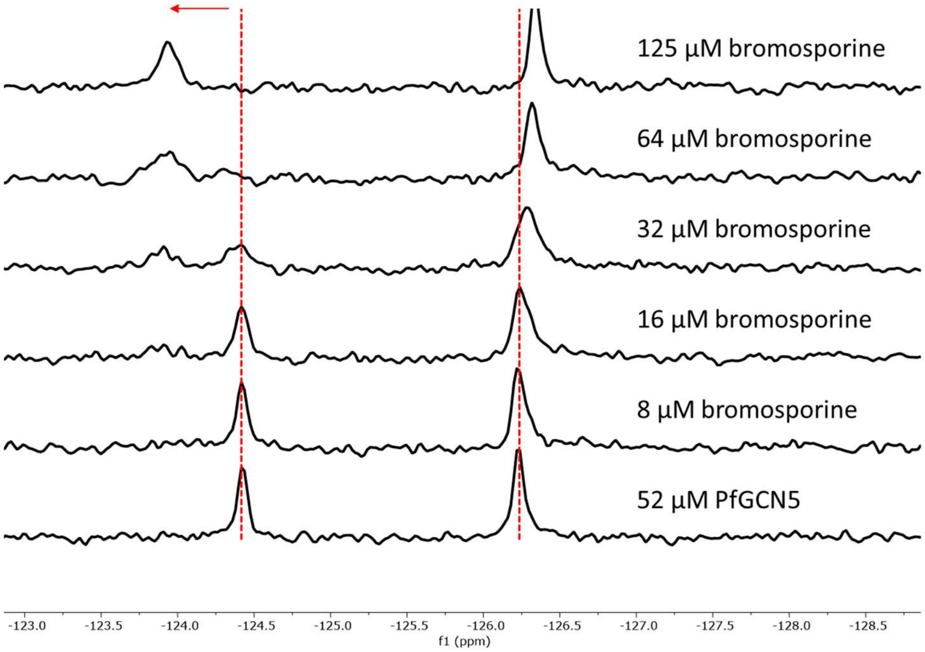

Figure S21. Titration of known pan-bromdomain ligand, bromosporine, with 5FW *Pf*GCN5. Slow exchange binding kinetics is observed for W1379 (W in the WPF shelf near the native binding site), consistent with a tight binding compound.

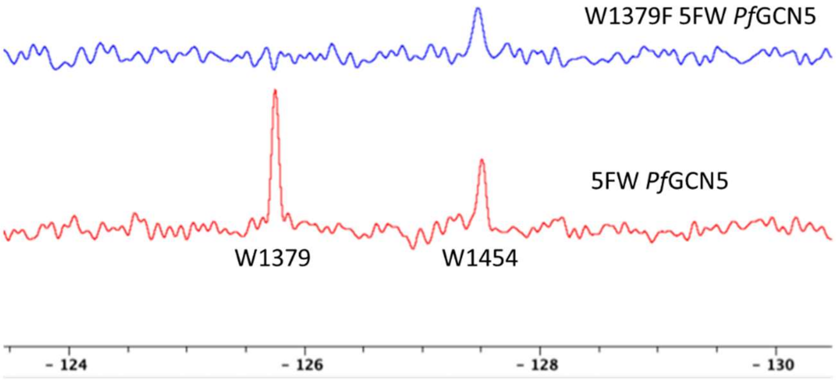

Figure S22. PrOF NMR overlay of 5FW-*Pf*GCN5 with W1379F 5FW-*Pf*GCN5.

Table S6. Chemical shifts and resonance width of 5FW labeled bromodomain when tested alone or in the presence of another 5FW labeled bromodomain.

Measured Protein	Second protein present	Chemical Shift of first 5FW resonance (ppm)	Full width at half height of first 5FW resonance 1 (HZ)	Chemical Shift of the second 5FW resonance (ppm)	Full width at half height of second 5FW resonance (HZ)	Chemical Shift of third 5FW resonance (ppm)	Full width at half height of third 5FW resonance (HZ)
PCAF	-	-125.12	41.9	-	-	-	-
PCAF	CECR2	-125.20 (0.08)	41.5 (0.4)	-	-	-	-
CECR2	-	-126.18	40.6	-	-	-	-
CECR2	PCAF	-126.205 (0.03)	43.5 (3.0)	-	-	-	
BPTF	-	-125.23	48.9	-	-	-	-
BPTF	PfGCN5	-125.19 (.05)	39.7 (9.2)	-	-	-	-
PfGCN5	-	-125.85	32.6	-127.65	40.4	-	-
PfGCN5	BPTF	-125.82 (0.03)	39.0 (6.9)	-127.60 (0.05)	43.2 (2.8)	-	-
BPTF	BRD4 (D1)	-125.21 (0.03)	40.0 (8.6)	-	-	-	-
BRD4 (D1)	-	-124.85	41.9	-125.48	42.0	-126.39	39.6
BRD4 (D1)	BPTF	-124.82 (0.03)	42.0 (0.5)	-125.48 (0.00)	41.1 (0.9)	-126.39 (0.00)	37.4 (2.2)

Average change in chemical shift from protein alone vs proteins tested together (ppm)	**0.04**
Average change in full width at half height from protein tested along to proteins tested together (Hz)	**4.31**

Change in chemical shift and change in the resonance width at half height from the protein tested alone to two proteins tested together is shown in parentheses.

1. Urick, A.K.; Hawk, L.M.L.; Cassel, M.K.; Mishra, N.K.; Liu, S.; Adhikari, N.; Zhang, W.; dos Santos, C.O.; Hall, J.L.; Pomerantz, W.C.K. Dual Screening of BPTF and Brd4 Using Protein-Observed Fluorine NMR Uncovers New Bromodomain Probe Molecules. *ACS Chem. Biol.* **2015**, *10*, 2246-2256, doi:10.1021/acschembio.5b00483.
2. Kozakov, D.; Grove, L.E.; Hall, D.R.; Bohnuud, T.; Mottarella, S.E.; Luo, L.; Xia, B.; Beglov, D.; Vajda, S. The FTMap family of web servers for determining and characterizing ligand-binding hot spots of proteins. *Nature Protocols* **2015**, *10*, 733-755, doi:10.1038/nprot.2015.043.
3. Brenke, R.; Kozakov, D.; Chuang, G.-Y.; Beglov, D.; Hall, D.; Landon, M.R.; Mattos, C.; Vajda, S. Fragment-based identification of druggable 'hot spots' of proteins using Fourier domain correlation techniques. *Bioinformatics (Oxford, England)* **2009**, *25*, 621-627, doi:10.1093/bioinformatics/btp036.
4. Kozakov, D.; Hall, D.R.; Chuang, G.-Y.; Cencic, R.; Brenke, R.; Grove, L.E.; Beglov, D.; Pelletier, J.; Whitty, A.; Vajda, S. Structural conservation of druggable hot spots in protein-protein interfaces. *Proc. Natl. Acad. Sci. U.S.A.* **2011**, *108*, 13528-13533, doi:10.1073/pnas.1101835108.
